# Transcriptomic analysis of *Penaeus monodon* in response to acute and chronic hypotonic stress

**DOI:** 10.3389/fvets.2024.1464291

**Published:** 2024-08-21

**Authors:** Jing Ji, Qiaohuang Wang, Shuigen Li, Yanting Chen, Jiexin Zhang, Hanxiu Yu, Jinzhen Xu, Miaomiao Li, Renhao Zheng, Nan Lin, Ziping Zhang

**Affiliations:** ^1^Fujian Agriculture and Forestry University, Fuzhou, China; ^2^Fujian Provincial Fisheries Technology Extension Center, Fuzhou, China; ^3^College of Marine Sciences, Fujian Agriculture and Forestry University, Fuzhou, China; ^4^Fisheries College, Jimei University, Xiamen, China; ^5^College of Animal Sciences, Fujian Agriculture and Forestry University, Fuzhou, China; ^6^State Key Laboratory of Mariculture Breeding, Fujian Agriculture and Forestry University, Fuzhou, China

**Keywords:** *Penaeus monodon*, hypotonic stress, transcriptome sequencing, bioinformatics analysis, differential gene expression

## Abstract

To investigate the different mechanisms of *Penaeus monodon* in response to acute and chronic hypotonic stress, RNA sequencing technology was employed to profile the gene expression patterns in the gill, hepatopancreas, and hemocyte at 0, 6, 48, and 72 h post acute hypotonic stress treatment (with salinity immediately decreased from 20 psu to 4 psu) and at 0, 2, 10, 15 days during chronic hypotonic stress treatment (with salinity gradually decreased from 20 psu to 4 psu). The control group (SC) was maintained at a constant salinity of 20 psu. Differentially expressed genes (DEGs) were identified, followed by further validation using real-time quantitative reverse transcription PCR (RT-qPCR). A total of 34,217 genes were expressed through sequencing. Compared with the control group, 8,503 DEGs were identified in the acute hypotonic stress group, comprising 3,266 up-regulated and 5,237 down-regulated genes. In the chronic hypotonic stress group, 8,900 DEGs were detected, including 3,019 up-regulated and 5,881 down-regulated genes. Gene Ontology (GO) functional annotation analysis indicated that DEGs were primarily enriched in biological processes such as cellular and metabolic processes, cellular components like membrane and other cellular components, and molecular functions including structural binding and catalytic activity. Kyoto Encyclopedia of Genes and Genomes (KEGG) pathway enrichment analysis indicated that DEGs were predominantly concentrated in five major pathways: metabolism, genetic information processing, environmental information processing, cellular processes, and biological systems. These pathways encompassed antigen processing and presentation, the NOD-like receptor signaling pathway, the Toll-like receptor signaling and cell apoptosis. The RT-qPCR validation of 11 DEGs (*hsp*70, *hsp*90, *nlrp*3, *mincle*, *nlrp*12, *tlr*4, *myd*88, *imd*, *casp*7, *casp*9 and *toll*) demonstrated that the trends observed in the quantitative results were consistent with those from the transcriptome analysis, thereby validating the reliability of transcriptome sequencing data. This study identified that hypotonic stress triggers physiological responses in *P. monodon* to both acute and chronic hypotonic conditions, offering valuable insights into the expression patterns of functional genes in the gills, hepatopancreas, and hemocytes of *P. monodon* under such stress. These findings provide foundational data and a theoretical basis for further research into the regulatory mechanism of *P. monodon* in response to hypotonic stress.

## Introduction

*Penaeus monodon* offers numerous advantages, such as economic benefits, growth characteristics, and nutritional value, making it an increasingly important species in aquaculture. Salinity is a crucial environmental factor affecting the cultivation of economically significant aquatic species. It can impact various aspects of marine crustaceans, including growth and survival (1), physiological activities and nutritional requirements (2), energy metabolism (3), and immunity (4). As a euryhaline species, *P. monodon* can survive across a wide salinity range, from 5 psu to 33 psu. Recent studies have demonstrated that under acute salinity stress, *Litopenaeus vannamei* can regulate osmotic ions through hyperglycemia (5). Furthermore, salinity stress has been shown to increase mortality in *L. vannamei* infected with white spot syndrome virus (WSSV), with the risk of infection escalating as salinity decreases from 35 psu to 10 psu (6). Similar findings have also been observed in *Fenneropenaeus indicus* (7). Liu et al. (8) revealed that under acute salinity stress, dopamine and 5-hydroxytryptamine can regulate free amino acids production, Na^+^/K^+^ pump activity, and the osmotic pressure regulation by glutamate dehydrogenase. Moreover, glutamate dehydrogenase can enhance the metabolism of free amino acids, particularly in the synthesis of arginine, proline and alanine. Studies on the immune activity and pathogenicity of *Vibrio harveyi* in *P. monodon* under acute hypotonic stress have shown that salinity affects the immune capacity and metabolic performance of *P. monodon*, increasing its susceptibility to viruses and enhancing the pathogenicity of viruses towards *P. monodon* (9). The immune capacity of *P. monodon* is reduced under both hypotonic and hypertonic conditions (9). Shekhar et al. (10, 11) studied the differential gene expression under chronic salinity stress in hypotonic and hypertonic conditions, concluding that these genes are involved in the regulatory mechanisms of adaptation to hypotonic stress.

Transcriptome sequencing technology is a molecular biology technique used to examine gene expression profiles in biological specimens. In recent years, transcriptome analysis has become a key tool for identifying differences in gene expression levels in various shrimp species under environmental stress. Notable examples include *L. vannamei* (12), *Palaemon gravieri* (13), and *Fenneropenaeus chinensis* (14). Qiao et al. (4) demonstrated that the addition of β-glucan can improve total antioxidant capacity (T-AOC), superoxide dismutase (SOD), and catalase (CAT) activities, thereby enhancing the antioxidant capacity of *L. vannamei* and reducing the damage caused by hypotonic stress. Farhadi et al. (15) reported the significant expression of various DEGs in *L. vannamei* under multiple stressors, including low salinity, nitrite exposure, low pH, and high pH. These DEGs include C-type lectin 2, anti-lipopolysaccharide factor 1 (*alf*1), acyl-coenzyme A oxidase 1-like (*acx*1), liver lectin-like, and hemolymph coagulation protein-like (*cp*).

Currently, research on the effects of salinity on *P. monodon* is primarily focused on salinity-related genes (16, 17) and acute immune responses (18). However, the specific effects of acute and chronic hypotonic stress on the physiological changes and gene expression in *P. monodon* remain largely unexplored. Therefore, this study aims to perform a transcriptome analysis of acute and chronic hypotonic stress in *P. monodon*. Such research is of great significance for disease prevention and the advancement of aquaculture technology.

## Materials and methods

### Experimental animals

The shrimps used in this experiment were provided by an aquaculture seedling farm in Zhangzhou, Fujian Province, with an average body length of 6.16 ± 1.12 cm and an average body weight of 4.56 ± 0.57 g, all healthy. Before the experiment, they were temporarily housed in a breeding barrel with a water temperature of 28.50 ± 2.0°C and salinity at 20 ± 1.0 psu. They were fed daily, with a water exchange of one-third of the volume with seawater every 3 days, and this acclimation feeding lasted for 5 days.

### Preliminary experiment

The shrimps were initially acclimated at a salinity of 20 psu for 5 days, followed by a gradual reduction of salinity to 15 psu, 10 psu, 7 psu, 5 psu, and 4 psu every 2 days. Samples were collected, and the mortality rates were recorded. As reported in the literature (19–21), *glut*, *nka*, and *ca* were identified as genes related to osmoregulation, and *gapdh* was employed as an internal control to evaluate the expression changes of these osmoregulation-related genes under hypotonic stress. The primers used for the tests are listed in Table 1.

**Table 1:** Primer sequences.

Gene name	Sequence (5′ to 3′)
*ca*	F: TCCCAGGAACAACTGGATGC
	R: AGAGAGGACATGGTGGCCTA
*glut*	F: CAAGGTGCCAGAGACCAAGAA
	R: ATCTGGCCCTACTTCCGTGT
*nka*	F: CCTGCCATTTCCCTTGCCTA
	R: AGCTTGTCGGTGAATGGGTT
*gapdh*	F: CGAGATGAAGCCCGAGAACA
	R: GCCTTCTCGATGGTGGTGAA

### Hypotonic stress experiments

The hypotonic stress experiment was conducted in two phases: acute and chronic hypotonic conditions. For the acute hypotonic experiment (Experiment J), seawater at 4 psu salinity was prepared, and 30 shrimps that had been pre-fed for 5 days were placed in each of the three buckets. The control group received an adequate amount of seawater at 20 psu salinity, with 30 shrimps (acclimated for 5 days) in each bucket. Samples were taken from three shrimps in each group at 6 h, 48 h, and 72 h, with gill, hepatopancreas, and hemocyte samples collected, using 20 psu salinity and the 0-h time point as control references throughout the experiment. In the chronic hypotonic experiment (Experiment M), 90 shrimps were transferred from the temporary holding buckets and evenly divided into three groups. They were placed in tanks containing pre-prepared seawater at a salinity of 20 psu. Fresh water was added daily to gradually reduce the salinity by 2 psu, reaching a final salinity of 4 psu over a period of 15 days. From the start reduction, three shrimps were sampled from each group on 2 days, 10 days, and 15 days, with gill, hepatopancreas, and hemocyte samples collected at each sampling time. Salinity levels of 20 psu and the 0-day time point served as controls.

### Sample collection and preparation

Gills and hepatopancreas were collected by removing the carapace of the head and thorax regions of the shrimps using sterilized scissors and tweezers. The dissected gills and hepatopancreas were then placed into cryostorage tubes containing RNAlater(Takara, Japan) and stored at −80°C. An appropriate amount of anticoagulant was prepared for hemocyte collection, and a 1 mL sterile syringe and a 2 mL sterile centrifuge tube were rinsed, leaving a sufficient amount of anticoagulant in the syringe. A shrimp was placed on a foam box containing crushed ice, and its surface was disinfected with an alcohol-soaked cotton ball. After disinfection, the shrimp was removed and gently blotted with a paper towel to remove excess liquid from its body surface. The shrimp’s head and chest were secured between the index and middle fingers, exposing the base of its last pair of pereiopods. The syringe’s needle was carefully inserted into the base of the fifth pair of pereiopods, and then the syringe’s plunger was slowly withdrawn to collect the hemolymph. After collection, the sample was centrifuged at 5,000 rpm at 4°C for 10 min to obtain the pellet. An appropriate amount of RNAlater (Takara, Japan) was added to the pellet, and the sample was stored at −80°C.

### Total RNA extraction, quantification and qualification

Total RNA from the samples was extracted using a standard Takara Kit (Takara, Japan), following the manufacturer’s protocol. RNA concentration and purity were assessed using a NanoDrop 2000 (Thermo Scientific, Massachusetts, United States). The integrity of the RNA was assessed using agarose gel electrophoresis, and the RNA quality number (RQN) value was determined using an Agilent 5300 bioanalyzer (Agilent Technologies, California, United States). Only high-quality RNA samples characterized by an OD_260/280_ ratio of 1.8 to 2.2, an OD_260/230_ ratio greater than or equal to 2.0, an RQN value of 6.5 or higher, a 28S: 18S rRNA peak intensity ratio of at least 1.0, and a quantity greater than 1 μg, were used to construct the sequencing library.

### Library preparation and Illumina sequencing

RNA purification, reverse transcription, library construction and sequencing were performed at Shanghai Majorbio Bio-Pharm Biotechnology Co., Ltd. (Shanghai, China) following the manufacturer’s instructions. The shrimp RNA-seq transcriptome library was prepared using Illumina^®^ Stranded mRNA Prep Kit (San Diego, CA), starting with 1 μg of total RNA. Initially, mRNA was isolated using the poly-A selection method with oligo(dT) beads, followed by fragmentation using a fragmentation buffer. Subsequently, double-stranded cDNA was synthesized using a SuperScript Double-Stranded cDNA Synthesis Kit (Invitrogen, CA) and random hexamer primers. Following the library construction protocol, the synthesized cDNA underwent end-repair, phosphorylation, and adapter ligation. Libraries were size-selected for cDNA target fragments of 300 bp using a 2% Low Range Ultra Agarose gel, followed by PCR amplification using Phusion DNA polymerase (NEB) for 15 cycles. After quantification with the Qubit 4.0 Fluorometer, the sequencing library was prepared on the NovaSeq X Plus platform (PE150) using the NovaSeq Reagent Kit (Illumina, United States).

### Transcriptome quality control and read mapping

The raw paired-end reads were trimmed and quality-controlled using FASTQ with default parameters. The cleaned reads were then aligned separately to the reference genome in orientation mode using HISAT2 software. The mapped reads of each sample were assembled using StringTie, employing a reference-based approach.

### Differential expression analysis and functional enrichment

To identify DEGs among different samples, the expression level of each transcript was calculated using the transcripts per million reads (TPM) method, with gene abundances quantified using RSEM. Differential expression analysis was performed using DESeq2 or DEGseq. DEGs with |log2FC| ≥1 and FDR <0.05 (DESeq2) or FDR <0.001 (DEGseq) were considered significantly differentially expressed genes. In addition, functional enrichment analysis, including GO and KEGG, was performed to identify DEGs that were significantly enriched in GO terms and metabolic pathways, with a Bonferroni-corrected *p*-value of less than 0.05 compared to the whole transcriptome background. GO functional enrichment and KEGG pathway analysis were conducted using Goatools and Python scipy software, respectively.

In this experiment, differences between the control group, the acute hypotonic stress group and the chronic hypotonic stress group were analyzed, and specific differences are summarized in Table 2.

**Table 2:** Comparison of samples from treatments and control.

Control group	Experimental group
SC_Gil	J6_Gil
SC_Gil	J48_Gil
SC_Gil	J72_Gil
SC_Gil	M2_Gil
SC_Gil	M10_Gil
SC_Gil	M15_Gil
SC_Hep	J6_Hep
SC_Hep	J48_Hep
SC_Hep	J72_Hep
SC_Hep	M2_Hep
SC_Hep	M10_Hep
SC_Hep	M15_Hep
SC_Hea	J6_Hea
SC_Hea	J48_Hea
SC_Hea	J72_Hea
SC_Hea	M2_Hea
SC_Hea	M10_Hea
J6_Gil	M2_Gil
J6_Hea	M2_Hea
J48_Hep	M2_Hep
J48_Gil	M2_Gil
J48_Hea	M2_Hea
J48_Hep	M10_Hep
J48_Gil	M10_Gil
J48_Hea	M10_Hea
J72_Hep	M15_Hep
J72_Gil	M15_Gil
J72_Hea	M15_Hea

Significance of comparison between acute and chronic hypotonic stress groups: J6 and M2 represent the initial time point of acute and chronic hypotonic stress experiments, respectively; M2 denotes 48 h mark in the chronic hypotonic stress period, while M10 signifies 48 h after the salinity has dropped to 4 psu during the chronic hypotonic stress period. J72 and M15 represent the later stages of the acute and chronic hypotonic stress experiments, respectively, indicating potentially weakened responses.

### Quantitative reverse transcription (qRT-PCR) validation

To verify the transcriptome sequencing results, 11 DEGs were randomly selected for quantitative real-time PCR. The *gapdh* was selected as an internal gene for reverse transcription template synthesis of the first chain as RT-qPCR. Primers were designed using the NCBI Primer-BLAST tool^1^ (Tables 1, 3), RT-qPCR was performed using PowerUP^™^ SYBR^™^ Green PCR Master Mix kit (Thermo Scientific, USA). The relative expression levels of each gene were calculated by detecting the cycle threshold (Ct) values when the target gene and internal reference gene reached the threshold. MicroAmp^®^ Optical 384-well plates were used with a total reaction value of 10 μL. The amplified reaction mixture contained 0.5 μL cDNA, 0.25 μL of 10 μM forward-primer, 0.25 μL of 10 μM reverse primer, 5 μL SYBR Green Real-time PCR Master Mix, and 4 μL nuclease-free water. The steps of the qPCR amplification reaction were as follows: UDG enzyme inactivation at 50°C for 2 min, predenaturation at 95°C for 2 min, followed by 40 cycles, respectively at 95°C for 15 s, 60°C for 15 s, and 72°C for 1 min. According to the Ct value measured by the system, the relative expression of each gene was calculated by $2^{-\Delta \Delta C_{T}}\left(\Delta \Delta C_{T}=\Delta C_{T\mathrm{treatment}}-\Delta C_{T\mathrm{control}}\right)$ and compared with the transcriptome data.

**Table 3:** RT-qPCR primer sequences.

Gene name	Sequence (5′ to 3′)
*hsp*70	F: AGTGAAATCGACAGCCGGAG
	R: CACGCTTGTTGTCGGTGAAG
*hsp*90	F: AGGCTCTTTCACCGTTCGTC
	R: AACGTACTCCGTCTGGTCCT
*nlrp*3	F: ACTGAGCAAGACCAGTGACG
	R: TGTTCTCCTTGTGCCGACAT
*mincle*	F: GCCAATGGAAGTGGCTGAAC
	R: CTCCTTCGTGAATGCCAGGT
*nlrp*12	F: ACTCACGAGCAAGGAAGCAA
	R: GACACATCGCCGACAGAGAA
*tlr*4	F: ACGGTTTCCTCAGCATGGTT
	R: GGGATTACCGGCCAAGTTGA
*myd*88	F: TGCTCATGCTCTCAGCGTAG
	R: TCAAGGGAGTGGCAGAAACT
*imd*	F: ACAACATAACAGGCGGCTCG
	R: AGGCATATCCTGGGGTTTGTG
*casp*7	F: AGCGATAAATTGGTGCGGCT
	R: AGCCTTCGTTCTCGCCATC
*casp*9	F: AGTTATCCCACCGAGACCCA
	R: GGTGTCGATTTCGTCCTGGT
*toll*	F: ACTGGTTCCCTGGAGCTTAC
	R: CTTGGCAGTGAGCTGTCTTG

## Results

### Preliminary experiment

**Figure 1:**
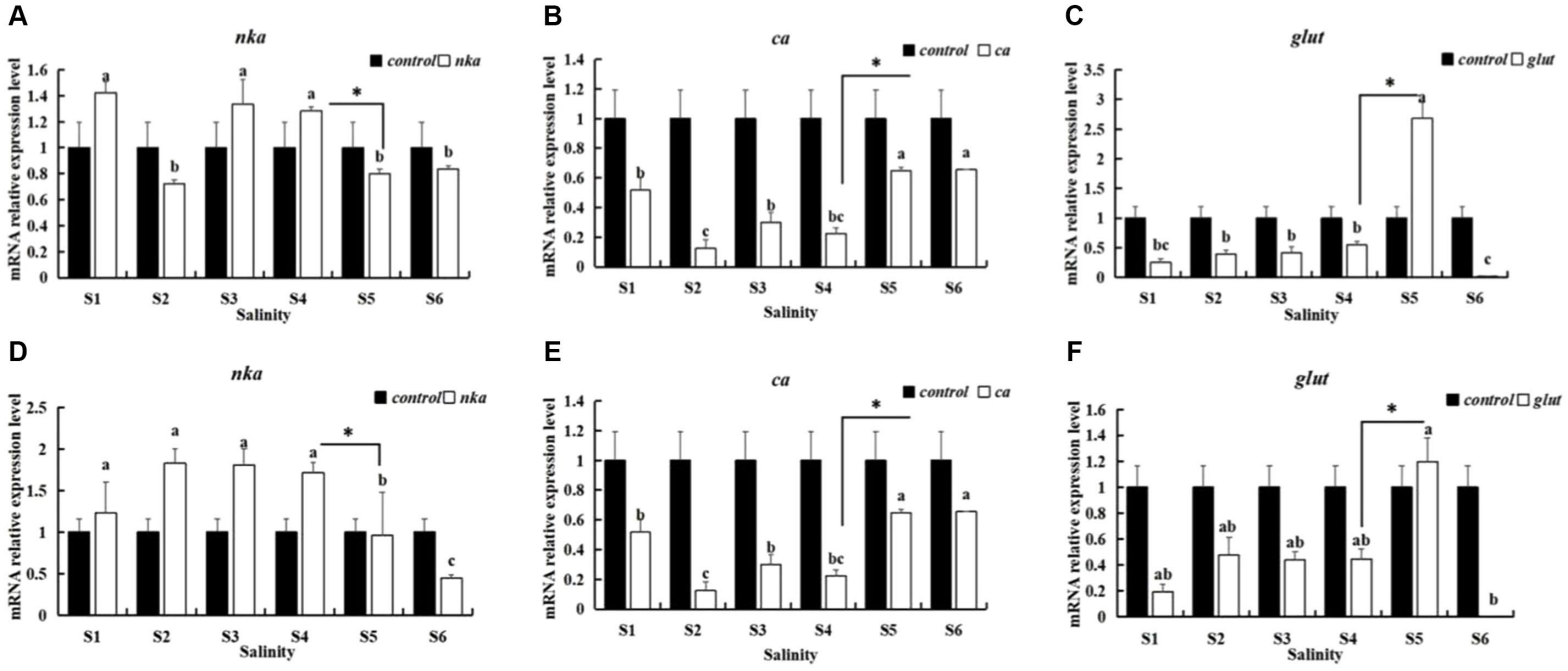
Expression of indicator genes in the gills and hepatopancreas of *P. monodon*. S1: 20 ‰ to 15 ‰, S2: 15 ‰ to 10 ‰, S3: 10 ‰ to 7 ‰, S4: 7 ‰ to 5 ‰, S5: 5 ‰ to 4 ‰, S6: 4 ‰ to 3 ‰. **(A–C)** Gills. **(D–F)** Hepatopancreas. **(A,D)** *nak*. **(B,E)** *ca*. (**C,F**) *glut*. “Control” indicates the expression of related genes expression under salinity at 20 psu. Lowercase letters indicate the significance between salinity groups (Duncan test, *p <* 0.05), and “*” indicates the significance between S4 and S5 (Duncan test, *p <* 0.05).

**Figure 2:**
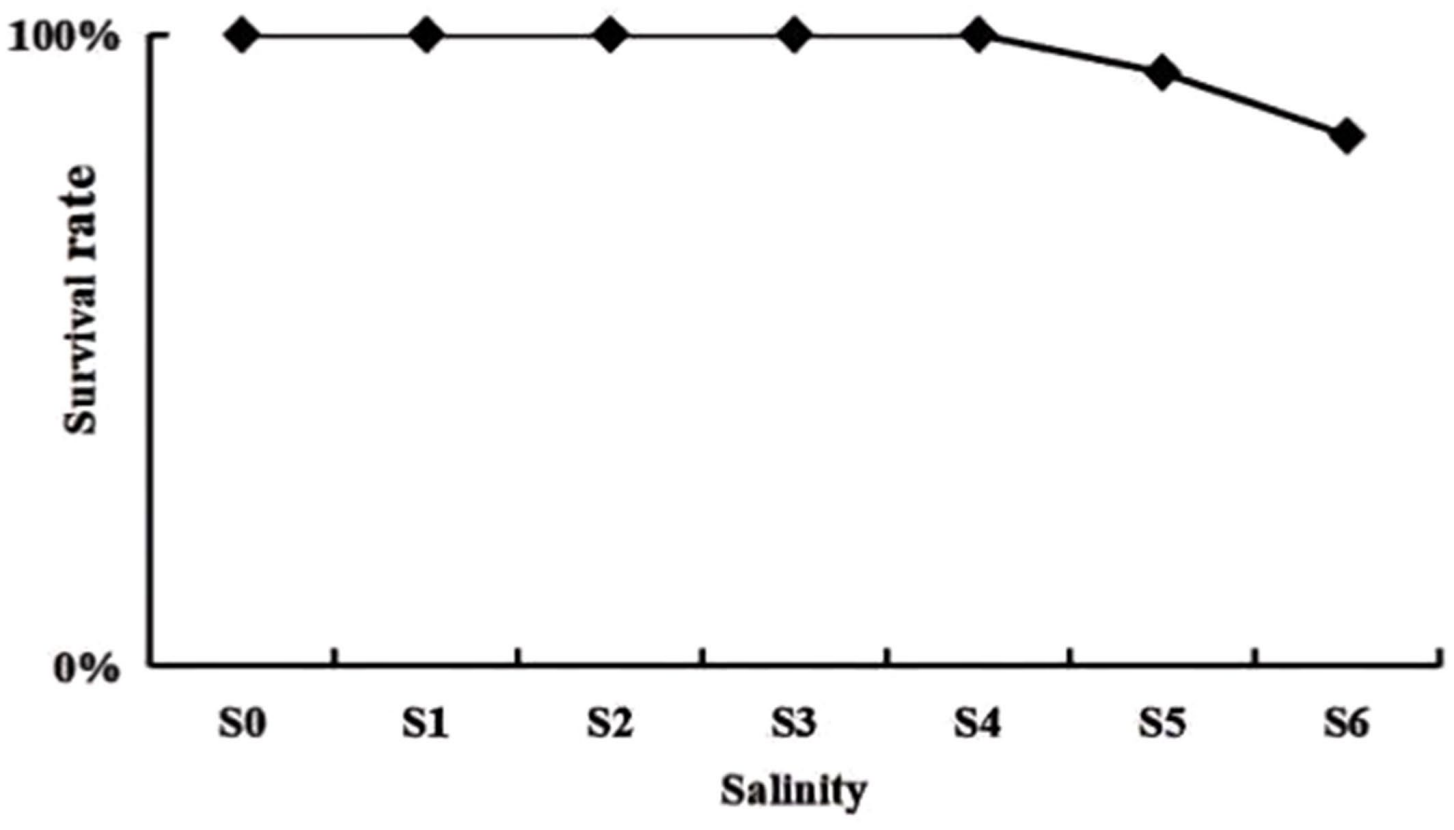
Survival of *P. monodon* in the preliminary experiment. S0: 20 ‰, S1: 20 ‰ to 15 ‰, S2: 15 ‰ to 10 ‰, S3: 10 ‰ to 7 ‰, S4: 7 ‰ to 5 ‰, S5: 5 ‰ to 4 ‰, S6: 4 ‰ to 3 ‰.

When the salinity decreased from 5 psu to 4 psu for the S5 group, the expression of *nka*, *ca* and *glut* in the gills and hepatopancreas were significantly up-regulated or down-regulated compared to the S4 group (Figure 1). This indicates that the salinity of 4 psu is a critical threshold that affects the growth and survival of *P. monodon* under hypotonic stress. In addition, in each salinity gradient of the preliminary experiment, a decrease from 5 psu to 4 psu coincided with the initial mortality observed in *P. monodon* (Figure 2). Consequently, a salinity of 4 psu was selected as the experimental salinity in this study.

### Illumina sequencing and *de novo* assembly

Supplementary Table S1 shows the results of sequencing data. A total of 3,444,112,332 clean reads were obtained through the NovaSeq X Plus platform (PE150), with a total length of 514,573,560,444 bp. The proportion of Q30 bases among samples exceeded 93.49%. Based on the selected reference genome sequence, String Tie or Cufflinks software was employed to assemble the mapped reads. It can be seen from Supplementary Figure S1 and Table 4 that the proportion of transcripts in the >1,800 bp segment was 39.75%, while the proportion of transcripts in the 1,001–1,800 bp segment was 21.03%.

**Table 4:** Statistics of assembly results.

Length	Number	Percent
0–200	3,023	3.99%
201–600	14,879	19.62%
601–1,000	11,844	15.61%
1,001–1,800	15,955	21.03%
>1,800	30,152	39.75%
Total	75,853	100%

### Functional classification and annotation

All genes and transcripts obtained by transcriptome assembly were aligned with six major databases (NR, Swiss-Prot, Pfam, EggNOG, GO, and KEGG), and a total of 22,548 Expressive Genes were annotated into the database (Supplementary Figure S2).

### Expression difference analysis

In this study, healthy shrimps cultured at a salinity of 20 psu served as the control group. Expression of differential genes in the gills, hepatopancreas, and hemocytes was assessed in both acute and chronic hypotonic stress groups. Specific statistical results are shown in Table 5. A Venn diagram analysis was performed on the distribution of differential genes in each phase, as depicted in Supplementary Figure S3.

**Table 5:** Statistics on the number of significant DEGs among gills, hepatopancreas and hemocytes.

Comparison between samples	All unigenes	Up-expressed unigenes	Down-expressed unigenes
SC_Gil_vs_J6_Gil_G	177	39	138
SC_Gil_vs_J48_Gil_G	146	48	98
SC_Gil_vs_J72_Gil_G	134	21	113
SC_Hep_vs_J6_Hep_G	126	64	62
SC_Hep_vs_J48_Hep_G	179	62	117
SC_Hep_vs_J72_Hep_G	56	21	35
SC_Hea_vs_J6_Hea_G	2,720	1,033	1,687
SC_Hea_vs_J48_Hea_G	2076	1,050	1,026
SC_Hea_vs_J72_Hea_G	2,889	928	1961
SC_Gil_vs_M2_Gil_G	140	5	135
SC_Gil_vs_M10_Gil_G	240	91	149
SC_Gil_vs_M15_Gil_G	445	215	230
SC_Hep_vs_M2_Hep_G	59	45	14
SC_Hep_vs_M10_Hep_G	235	140	95
SC_Hep_vs_M15_Hep_G	568	246	322
SC_Hea_vs_M2_Hea_G	2052	668	1,384
SC_Hea_vs_M10_Hea_G	2,697	837	1860
SC_Hea_vs_M15_Hea_G	2,464	772	1,692

Table 5 reveals a total of 17,403 DEGs in the gill, hepatopancreas, and hemocyte samples of *P. monodon* in response to acute and chronic hypotonic stress. Among these, 6,285 (36.11%) were up-regulated, while 11,118 (63.89%) were down-regulated. Specifically, there were 8,503 DEGs under acute hypotonic stress and 8,900 DEGs under chronic hypotonic stress. In gill samples, 1,282 DEGs were identified, with 419 (32.68%) up-regulated and 863 (67.32%) down-regulated under both stresses. Hepatopancreas samples exhibited 1,223 DEGs, with 578 (47.26%) up-regulated and 645 (52.74%) down-regulated. Hemocyte samples showed 14,898 DEGs, with 5,288 (35.49%) up-regulated and 9,610 (64.51%) down-regulated.

### Function annotations

#### COG annotations

**Figure 3:**
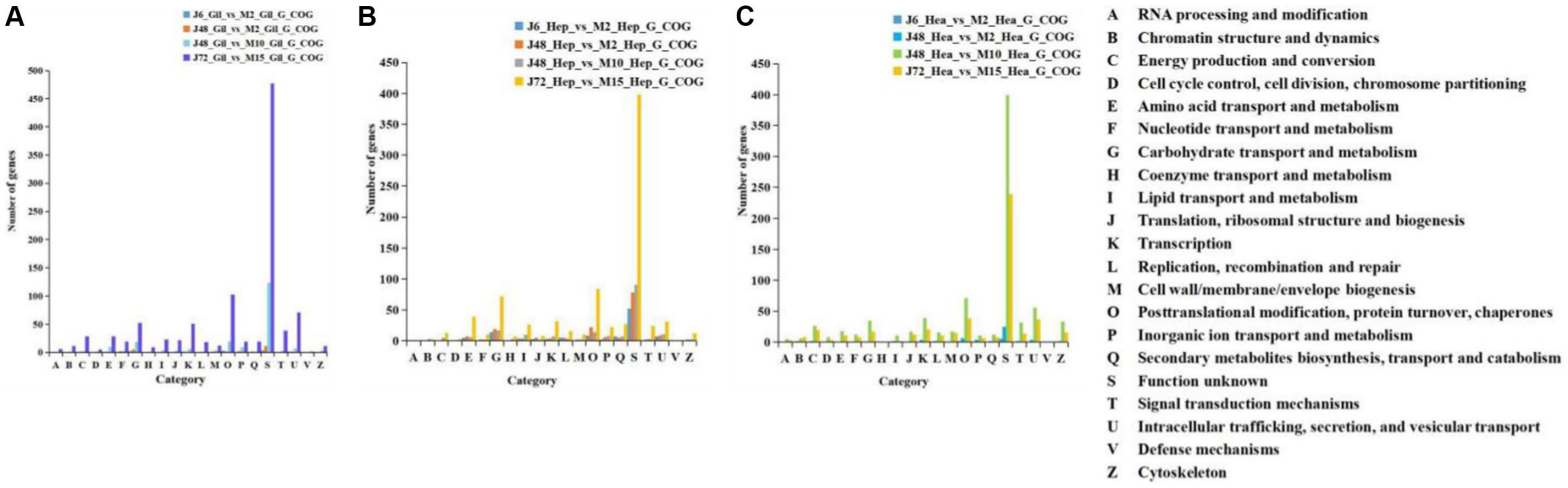
COG comparison of acute and chronic hypotonic stress in gills, hepatopancreas and hemocytes of *P. monodon*. **(A)** Gills. **(B)** Hepatopancreas. **(C)** Hemocytes.

Figure 3 illustrates COG functional annotations for *P. monodon* under acute and chronic hypotonic stress. The most annotated COG category is S (unknown function), indicating that a substantial number of proteins have yet to determine their functions. Among those with characterized functions, a significant number of DEGs were observed in the gill, hepatopancreas, and hemocyte samples of *P. monodon* under acute hypotonic stress for 72 h and chronic hypotonic stress for 15 days, mainly associated with protein modification and transport.

#### GO function annotations

**Figure 4:**
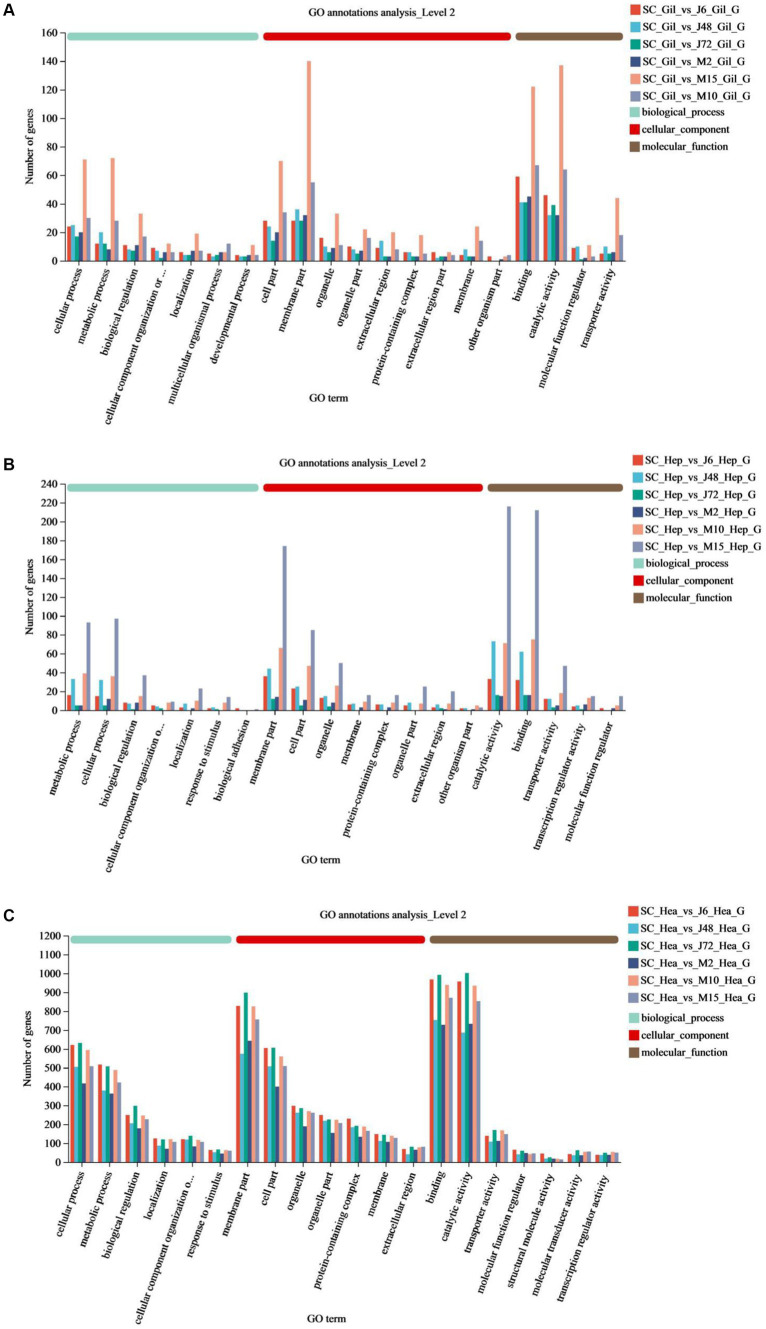
GO annotations of acute and chronic hypotonic stress in *P. monodon*. **(A–C)** Show the GO annotation of gills, hepatopancreas and hemocytes under acute hypotonic stress for 6 h, 48 h and 72 h, as well as chronic hypotonic stress for 2 days, 10 days and 15 days.

Table 6 and Figure 4 display the GO function annotations for a total of 17,403 DEGs, with distribution as follows: gills (1,282), hepatopancreas (1,223), and hemocytes (14,898), in the comparison groups. These annotations encompass three categories: Biological process (BP), cellular component (CC) and molecular function (MF). In the BP category, cellular processes were predominant in the gill group, while metabolic processes were predominant in the hepatopancreas group, and both cells and metabolic processes were prevalent in the hemocytes group. Regarding the CC category, gills, hepatopancreas, and hemocytes were all membrane parts. In the MF category, structural binding function and catalytic activity function were the most abundant across all three tissues. In addition, the biological process categories included some key genes related to the regulation of the immune system. These enriched genes may play a key role in the response to hypotonic stress in *P. monodon*.

**Table 6:** GO function annotation statistics table.

Intersample comparison	Biological process	Cellular component	Molecular function	Total differentially expressed genes
SC_Gil_vs_J6_Gil_G	16	12	10	1,282
SC_Gil_vs_J48_Gil_G				
SC_Gil_vs_J72_Gil_G				
SC_Gil_vs_M2_Gil_G				
SC_Gil_vs_M10_Gil_G				
SC_Gil_vs_M15_Gil_G				
SC_Hep_vs_J6_Hep_G	14	14	12	1,223
SC_Hep_vs_J48_Hep_G				
SC_Hep_vs_J72_Hep_G				
SC_Hep_vs_M2_Hep_G				
SC_Hep_vs_M10_Hep_G				
SC_Hep_vs_M15_Hep_G				
SC_Hea_vs_J6_Hea_G	21	16	12	14,898
SC_Hea_vs_J48_Hea_G				
SC_Hea_vs_J72_Hea_G				
SC_Hea_vs_M2_Hea_G				
SC_Hea_vs_M10_Hea_G				
SC_Hea_vs_M15_Hea_G				

### GO functional enrichment and KEGG enrichment analysis

#### GO functional enrichment

GO functional enrichment analysis was conducted to determine the functional properties of the DEGs and their products in each group under acute and chronic hypotonic stress. The results revealed that the DEGs were mainly divided into three categories: BP, MF, and CC. The GO classification diagram and specific information are shown in Supplementary Figures S4–S6. In gills, significantly enriched BP included the molting cycle process, proline catabolism, etc. MF encompassed N-acetyl-β-D-galactosaminidase activity, proton transport ATPase activity, and ion transmembrane transport activity, etc. CC comprised V-type proton transport ATPase, among others. In the hepatopancreas, significantly enriched BP included lipid metabolism, amino acid biosynthesis, catabolism, etc. MF encompassed amylase activity and oxidoreductase activity. In hemocytes, significantly enriched BP included RNA processing, redox processes, innate immune response activation of cell surface receptor signaling pathways, immune response regulation signaling pathways, etc. MF encompassed pattern recognition receptor activity, redox activity, Transport activity, etc. CC is comprised of mitochondria, mitochondrial membrane, organelle inner membrane, etc.

#### KEGG enrichment analysis

KEGG pathway analysis revealed 3,838 DEGs, with distribution as follows: gills (407), hepatopancreas (466), and hemocytes (2,965), which were annotated to the KEGG library, involving 275, 282 and 338 pathways, respectively. Among these, 5 main primary classification pathways exhibited significant enrichment, including metabolism, genetic information processing, environmental information processing, cellular processes, and biological systems. In the secondary pathway classification, for example, arginine and proline metabolism pathway, amino sugar and ribose metabolism pathway, mTOR signaling pathway, antigen processing and presentation pathway, RIG-I-like receptor signaling pathway, IL-17 signaling pathway and the Toll-like receptor signaling pathway-related genes were significantly enriched in these metabolic pathways. Detailed results are provided in Tables 7, 8.

**Table 7:** KEGG enrichment pathways.

Pathway ID	Pathway-description	Category
map04722	Neurotrophin signaling pathway	Nervous system
map04721	Synaptic vesicle cycle	Nervous system
map04728	Dopaminergic synapse	Nervous system
map04726	Serotonergic synapse	Nervous system
map04724	Glutamatergic synapse	Nervous system
map00260	Glycine, serine and threonine metabolism	Amino acid metabolism
map04110	Cell cycle	Cell proliferation and apoptosis
map04214	Apoptosis—fly	Cell proliferation and apoptosis
map04210	Apoptosis	Cell proliferation and apoptosis
map00190	Oxidative phosphorylation	Energy metabolism
map04612	Antigen processing and presentation	Immune system
map04623	Cytosolic DNA-sensing pathway	Immune system
map04621	NOD-like receptor signaling pathway	Immune system
map04625	C-type lectin receptor signaling pathway	Immune system
map04624	Toll and Imd signaling pathway	Immune system
map04622	RIG-I-like receptor signaling pathway	Immune system
map04620	Toll-like receptor signaling pathway	Immune system
map04664	Fc epsilon RI signaling pathway	Immune system
map04610	Complement and coagulation cascades	Immune system
map04657	IL-17 signaling pathway	Immune system
map00140	Steroid hormone biosynthesis	Lipid metabolism
map00561	Glycerolipid metabolism	Lipid metabolism
map00591	Linoleic acid metabolism	Lipid metabolism
map00230	Purine metabolism	Nucleotide metabolism
map00240	Pyrimidine metabolism	Nucleotide metabolism
map04630	JAK-STAT signaling pathway	Signal transduction
map04151	PI3K-Akt signaling pathway	Signal transduction
map04668	TNF signaling pathway	Signal transduction
map04150	mTOR signaling pathway	Signal transduction
map04668	TNF signaling pathway	Signal transduction
map04150	mTOR signaling pathway	Signal transduction
map04350	TGF-beta signaling pathway	Signal transduction
map04064	NF-kappa B signaling pathway	Signal transduction
map04064	NF-kappa B signaling pathway	Signal transduction
map04024	cAMP signaling pathway	Signal transduction
map04020	Calcium signaling pathway	Signal transduction
map03008	Ribosome biogenesis in eukaryotes	Translation
map04142	Lysosome	Transport and catabolism
map04136	Autophagy—other	Transport and catabolism

**Table 8:** Immune-related differentially expressed genes and their KEGG enrichment pathways.

Gene-ID	Gene-description	KEGG pathways
LOC119574143	Heat shock 70 kDa protein cognate 4-like	Antigen presentation and processing
LOC119590681	Heat shock protein HSP 90-alpha-like	Antigen presentation and processing
LOC119591691	Uncharacterized, transcript variant X1	C-type lectin receptor signaling pathway
LOC119582471	Calmodulin-like, transcript variant X2	C-type lectin receptor signaling pathway
LOC119590800	C-type lectin domain family 17, member A-like	C-type lectin receptor signaling pathway
LOC119590140	E3 ubiquitin-protein ligase Mdm2-like, transcript variant X2	C-type lectin receptor signaling pathway
LOC119587731	Uncharacterized	NOD-like receptor signaling pathway
LOC119591691	Uncharacterized transcript variant X1	NOD-like receptor signaling pathway
LOC119579524	Myeloid differentiation primary response protein MyD88-like, transcript variant X1	Toll-like receptor signaling pathway
LOC119599327	Stress-activated protein kinase JNK-like	Toll-like receptor signaling pathway
LOC119575137	Oplophorus-luciferin 2-monooxygenase non-catalytic subunit-like	Toll-like receptor signaling pathway
LOC119583060	Toll	Toll-like receptor signaling pathway
LOC119591291	Receptor-interacting serine/threonine-protein kinase 1-like, transcript variant X2	Toll and IMD signaling pathways
LOC119591492	Transcription factor kayak-like	Toll and IMD signaling pathways
LOC119597839	Caspase-1-like, transcript variant X1	Apoptosis
LOC119574498	Caspase-1-like	Apoptosis
LOC119583694	Caspase-13-like, transcript variant X1	RIG-I like receptor signaling pathway
LOC119598503	Serine/threonine-protein kinase mTOR-like, transcript variant X1	mTOR signaling pathway
LOC119593982	Nuclear factor NF-kappa-B p105 subunit-like	IL-17 signaling pathway

### Quantitative real-time PCR validation and expression pattern analysis

To test the reliability of transcriptome sequencing results, 11 DEGs (*hsp*70, *hsp*90, *nlrp*3, *mincle*, *nlrp*12, *tlr*4, *myd*88, *imd*, c*asp*7, *casp*9, *toll*) involved in immune and apoptosis-related signaling pathways were selected for qPCR validation analysis. Quantitative results and transcriptome analysis results are presented in Supplementary Figures S7–S9. The expression trends of these 11 DEGs, as determined by RT-qPCR validation, were found to be in general agreement with those from the transcriptome sequencing. Although the quantitative results of some genes were inconsistent with the sequencing results, the general trend was consistent. Consequently, the RT-qPCR results confirmed the credibility of the data obtained in this study. It can be seen from the results that after acute hypotonic stress, the expression levels of most genes increased with the increase of stress time, although a few genes showed decreased expressions. Under chronic hypotonic stress, the expression levels of most genes increased with the increase of hypotonic stress time, and the gene expression levels showed a downward trend. However, there were instances where the expression of some genes increased.

### Expression analysis of immune-related genes

**Figure 5:**
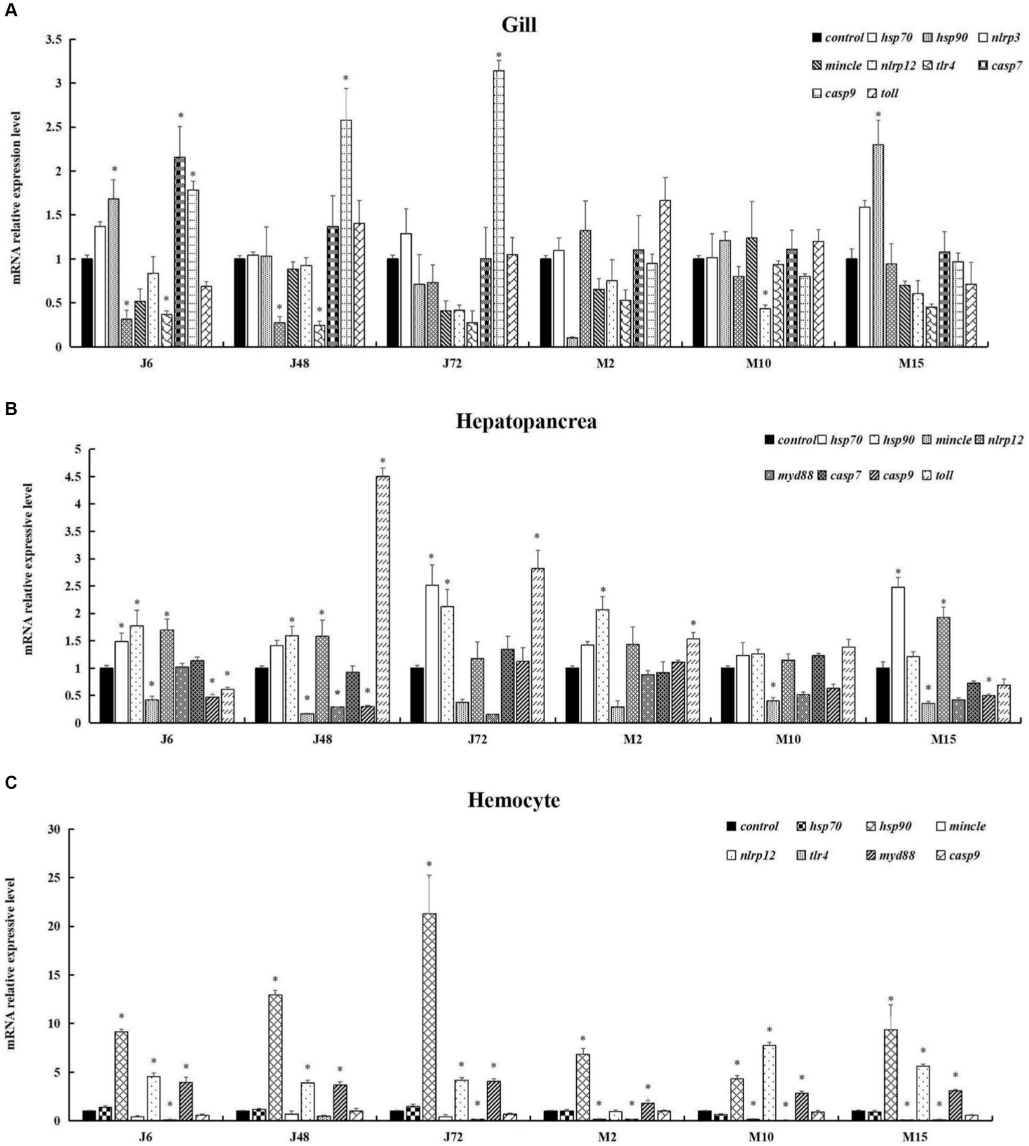
Expression of immune and apoptosis-related genes in *P. monodon* under acute and chronic hypotonic stress. The data in the figure are the average value and standard error. The ordinate represents the relative expression of RT-qPCR for each gene, and the “*” symbol on the column indicates the significance of the expression of different genes in the same group at the same time (Duncan test, *p* < 0.05). **(A)** Gills. **(B)** Hepatopancreas. **(C)** Hemocytes. “Control” indicates the expression of related genes expression under salinity at 20 psu. J6, J48, and J72 represent 6 h, 48 h, and 72 h under acute hypotonic stress, and M2, M10, and M15 represent 2 days, 10 days, and 15 days under chronic hypotonic stress, respectively.

As depicted in Figure 5, fluorescence quantitative amplification was performed on genes related to antigen presentation and processing, the Toll pathway, apoptosis, Toll and IMD pathways, C-type lectin pathway and other signaling pathways in the gills, hepatopancreas, and hemocytes of *P. monodon* under both acute and chronic hypotonic stress. In gills, after 6 h of acute hypotonic stress, the expression levels of *hsp*90, *casp*7, and *casp*9 were significantly up-regulated compared with the control group (*p* < 0.05); *cas*9 remained significantly up-regulated at 48 h and 72 h (*p* < 0.05). After chronic hypotonic stress, only the 15 days group exhibited a significant upregulation in *hsp*90 repression compared to the control group (*p* < 0.05). The expression levels of *nlrp*3 and *tlr*4 were significantly down-regulated at 6 h and 48 h after acute hypotonic stress (*p* < 0.05), while the expression levels of *hsp*90 and *nlrp*12 were significantly down-regulated at 2 days and 10 days after chronic hypotonic stress (*p* < 0.05).

In the hepatopancreas, after 6 h of acute hypotonic stress, the expression levels of *hsp*70, *hsp*90, and *nlrp*12 were significantly up-regulated (*p* < 0.05); at 48 h, *hsp*90, *nlrp*12, and *toll* were significantly up-regulated (*p* < 0.05); and at 72 h, *hsp*70, *hsp*90, and *toll* were significantly up-regulated (*p <* 0.05). After 2 days of chronic hypotonic stress, *hsp*90 and *toll* showed significant upregulation (*p <* 0.05); and after 15 days, *hsp*70 and *nlrp*12 were significantly up-regulated (*p* < 0.05). Conversely, at 6 h after acute hypotonic stress, *mincle*, *casp*9, and *toll* exhibited significant downregulation (*p* < 0.05). Similarly, *mincle* showed significant downregulation at 10 days and 15 days after chronic hypotonic stress (*p* < 0.05).

In hemocytes, significant up-regulation was observed in the expression levels of *hsp*90, *nlrp*12 and *myd*88 at 6 h, 48 h, and 72 h after acute hypotonic stress (*p* < 0.05). Following chronic hypotonic stress, significant up-regulation was observed in the expression levels of *hsp*90, *myd*88 at 2 days, while *hsp*90, *nlrp*12, and *myd*88 were significantly up-regulated at 10 days and 15 days. Conversely, at 6 h and 72 h after acute hypotonic stress, the expression level of *tlr*4 was significantly down-regulated (*p* < 0.05). Similarly, the expression levels of *mincle* and *tlr*4 were significantly down-regulated at 2 days, 10 days, and 15 days after chronic hypotonic stress (*p* < 0.05). No significant changes were observed in other genes compared with the control group in each phase (*p* > 0.05).

## Discussion

Transcriptome analysis technology or RNA sequencing stands out as a vital tool in omics research, which can provide a deeper insight into complex physiological pathways, including immune response, substance synthesis and metabolism, osmotic pressure regulation, growth and development. When aquatic animals’ gills are stimulated by the external environment or invaded by microorganisms, immune genes are activated to activate signaling pathways to initiate immune regulation. As the central organ for arthropod immunity and metabolism, the hepatopancreas plays a pivotal role in resisting pathogens, adapting to external changes and maintaining overall health (22, 23). Hemocytes, rich in immune factors and osmotic regulators, serve as crucial research materials. Moreover, these three components exhibit close interrelations when gills, hepatopancreas, and hemocytes play osmotic regulation and immune regulation under hypotonic stress. Hence, it is justifiable to perform transcriptomic analysis of total RNA obtained from gills, hepatopancreas, and hemocytes in the study.

Through KEGG and GO enrichment analyses, combined with transcriptome functional annotation, the genes (*hsp*70, *hsp*90, *nlrp*3, *mincle*, *nlrp*12, *tlr*4, *myd*88, *imd*, *casp*7, *casp*9 and *toll*) related to immunity and apoptosis of *P. monodon* after hypotonic stress were found to be differentially regulated in response to changes in salinity. Moreover, RT-qPCR experiments further verified and analyzed the immune alterations in *P. monodon* after hypotonic stress under different conditions. These results showed that different immune factors played roles during the immune process at different times. These findings are similar to the research by Guo et al. (24), which demonstrated that when *Cherax quadricarinatus* was infected with *Aeromonas veronii*, all humoral immune factors tested exhibited different activities over the course of infection. Furthermore, many immune-related signaling pathways were discovered, including antigen processing and presentation, NOD-like receptor signaling pathway, Toll signaling pathway, and apoptosis, among others. The results of the transcriptomic data from this study provided ample evidence that both acute and chronic hypotonic stress can trigger an immune response.

In this study, the genes associated with antigen presentation and processing pathways were *hsp7*0 and *hsp*90. In the gills of *P. monodon*, the expression trend of *hsp*90 gradually decreased under acute hypotonic stress, while under chronic hypotonic stress, it gradually increased, showing a contrasting trend. This might suggest that under acute hypotonic stress, differential genes are primarily involved in immune regulation, while during chronic hypotonic stress, osmotic regulation takes precedence. Additionally, compared to the control group, the expression of *hsp*90 decreased at 48 h compared to 6 h under acute hypotonic stress, and it also decreased at 2 days (equivalent to 48 h) under chronic hypotonic stress, possibly indicating that the protective mechanism of *hsp*90 in the short term under both acute and chronic hypotonic stress is similar. However, the *hsp*90 was significantly down-regulated under chronic hypotonic stress. The mRNA synthesis of *HSPs* in *L. vannamei* continued to decrease under hypoxic conditions, mainly due to mitochondrial damage and the slow response of the shrimp under hypoxic conditions (25). Therefore, there may be cellular damage in *P. monodon* at 2 days of hypotonic stress. In the hepatopancreas of *P. monodon*, *hsp*70 was significantly expressed under acute hypotonic stress compared to the control group in most time phases and only at 2 days and 15 days under chronic hypotonic stress, but *hsp*90 was significantly expressed in acute and chronic hypotonic stress. This might reveal that similar regulatory mechanisms may be at play in antigen presentation and processing under both acute and chronic hypotonic stress. Furthermore, in hemocytes, the *hsp*90 in each time phase of both acute and chronic hypotonic stress was significantly up-regulated, while there was no significant change of *hsp*70 gene in the gills and hemocytes, suggesting that the main regulation site of *hsp*70 might be the hepatopancreas. Conversely, most of the changes in *hsp*90 expression in gills, hepatopancreas and hemocytes were significant and mostly up-regulated. This may reveal that *hsp*90 plays a more prominent regulatory role than *hsp*70 in the antigen presentation processing pathway of *P. monodon* under hypotonic stress. For instance, after the infection of *Artemia franciscana* with *Gymnodinium catenatum*, both *hsp*70 and *hsp*90 were also up-regulated (26), mirroring the results of this experiment. The heightened expression of *hsp* may induce protein synthesis modification with immune function, eliminate chemical or foreign substances that damage cells, and enhance cell tolerance *in vivo*, thereby achieving the purpose of protecting cells. In *L. vannamei*, the expression of *hsp*70 and *hsp*90 increased at high temperatures, mainly due to accelerated metabolism, which generates sufficient energy for the synthesis of related proteins (25, 27). Similarly, in this experiment, the heightened expression of *hsp*70 and *hsp*90 under hypotonic stress may also be attributed to the accelerated metabolism of *P. monodon* under hypotonic stimulation, resulting in increased energy production, part of which is used for immune regulation and part for osmotic regulation.

Throughout the research, the genes related to the C-type lectin receptor signaling pathway are *nlrp*3 and *mincle*. In the gills of *P. monodon*, the expression of the *nlrp*3 was significantly down-regulated under acute hypotonic stress compared to the control group, while the expression levels of these two genes did not change significantly under chronic hypotonic stress. In the hepatopancreas of *P. monodon*, the expression of *mincle* was significantly down-regulated at 10 days and 15 days compared with the control group, and the expression of *mincle* in hemocytes of 15 days was significantly down-regulated compared with the control group. These findings suggest that the expression of genes related to the C-type lectin receptor signaling pathway genes in gills, hepatopancreas and hemocytes is inhibited under hypotonic stress. This may also indicate that the immunity of *P. monodon* at this time is reduced, and the immune regulation of this pathway may not follow the same pattern as that of healthy shrimp during bacterial infections or virus outbreaks. Consequently, when cultivating *P. monodon* in low-salinity environments, it becomes imperative to eradicate pathogens, regularly monitor whether the microorganisms in the water are qualified, and implement precautionary measures to maintain the health of the shrimp population. Nutrients such as probiotics and prebiotics that enhance shrimp immunity can also be supplemented in the water. The *nlrp*3 can be activated by external stress and induce downstream IL-1β. The expression level of *nlrp*3 under chronic hypotonic stress was generally higher than that under acute hypotonic stress, possibly indicating that *nlrp*3 induces chronic inflammation in shrimp under chronic hypotonic stress. *mincle* is a macrophage-induced C-type lectin gene that localizes to dead cells and surrounding macrophages to accelerate cell death. However, it inhibits the body’s ability to eliminate dead cells, leading to chronic inflammation. *mincle* was predominantly down-regulated in this study, and its activity was inhibited. This may suggest that under hypotonic stress, the body mainly removes dead cells in a timely manner. While research on *mincle* in crustaceans such as shrimps and crabs is limited, studies in mice have shown that *mincle* contributes to persistent inflammation in acute kidney injury (28, 29).

Across the scope of this study, the genes associated with the NOD-like receptor signaling pathway include *nlrp*3 and *nlrp*12, both of which can trigger downstream production of IL-1β and IL-18, leading to inflammation. In the gills of *P. monodon*, the expression of *nlrp*3 was significantly down-regulated at 6 h and 48 h of acute hypotonic stress compared to the control group, while the expression of the *nlrp*12 was significantly down-regulated under chronic hypotonic stress at 10 days compared to the control group. In the hepatopancreas of *P. monodon*, the *nlrp*12 was significantly up-regulated under acute stress at 6 h and 48 h compared to the control group, and it was significantly up-regulated at 15 days under chronic hypotonic stress. Additionally, compared to the control group, the *nlrp*12 in most time phases of acute and chronic hypotonic stress in the hemocytes was significantly up-regulated. These findings may suggest that the NOD-like receptor signaling pathway primarily functions in hemocytes, followed by the hepatopancreas, which is inhibited in the gills. *LvNLRPL1*, a novel NOD-like receptor gene found in *L. vannamei*, was also highly expressed in hemocytes, and its up-regulation enhances the antibacterial ability (30). Under bacterial infection, *LvNLRPL1* exerts an inhibitory effect on blood cell apoptosis by inhibiting the expression of caspases. Similarly, the up-regulation of *nlrp*12 in hemocytes may also inhibit apoptosis and enhance the immunity of blood cells, thereby protecting the organism against adverse external environments.

In this research, the Toll-like receptor signaling pathway-related genes include *toll*, *tlr*4, *myd*88. In the gills of *P. monodon*, the expression of *tlr*4 was significantly down-regulated under acute hypotonic stress compared to the control group, while the expression levels of all three genes did not change significantly under chronic hypotonic stress. In the hepatopancreas of *P. monodon*, the expression of *toll* was significantly down-regulated at 6 h under acute hypotonic stress, and it was significantly up-regulated at 48 h and 72 h compared to the control group, showing an upward trend. Under chronic hypotonic stress, the expression was only significantly up-regulated at 2 days. In hemocytes, the *myd*88 was significantly up-regulated in most time phases under acute hypotonic stress and 15 days under acute hypotonic stress compared to the control group. It may reveal that the three genes of *toll*, *tlr*4, *myd*88 in the Toll-like receptor signaling pathway exert their effects at different sites. This could suggest that the Toll-like receptor signaling pathway coordinates the immune regulation genes in gills, hepatopancreas, and hemocytes, indicating a collaborative regulation. The *toll* was highly expressed in the hepatopancreas and hemocytes under hypotonic stress, which aligns with similar findings with regard to *EcToll* expression in other shrimp species, such as the *Exopalaemon carinicauda* (31).

As observed in this study, the genes related to the apoptosis pathway are *casp*7 and *casp*9. In the gills of *P. monodon*, *casp*7 and *casp*9 were significantly up-regulated under acute hypotonic stress compared to the control group. However, *casp9* expression did not change significantly under chronic hypotonic stress. Conversely, in the hepatopancreas of *P. monodon*, the *casp*9 was significantly down-regulated under chronic hypotonic stress compared to the control group. The expression levels of *casp*7 and *casp*9 in the hemocytes did not change significantly, which may reveal a high rate of apoptosis in the gill tissue of *P. monodon* under acute hypotonic stress. This could be attributed to the initial exposure of the gill to external hypotonic stress, causing severe cell damage. However, the downregulation or insignificant changes in *casp*7 and *casp*9 expression under chronic hypotonic stress may suggest that *P. monodon* adapts to prolonged hypotonic stress by reducing the apoptosis rate and prolonging the cell lifespan to conserve energy and maintain survival. This adaptive strategy contrasts with the observations of increased apoptosis rate in hemocytes in *L. vannamei* and *S. paramamosain* infected with bacteria (32, 33). These differences may stem from distinct apoptotic regulation activated by hypotonic stress and pathogen invasion.

In the signal enrichment pathway analysis of this study, it was found that other signaling pathways were also involved in the signal enrichment pathway analysis. It was also discovered that neural regulation was also involved, such as dopaminergic synapses, serotonergic synapses, and glutamatergic synapses. Neuropeptides such as dopamine generate melanin through a series of catalytic reactions that undergo redox reactions (34, 35). Furthermore, pathways such as lysosomes and phagosomes are also activated under hypotonic stress in *P. monodon*, assisting in osmotic regulation and immune regulation, clearing harmful substances, and reducing bodily damage.

## Conclusion

This study revealed the disparities in the osmotic regulation of *P. monodon* under acute and chronic hypotonic stress, representing a complex physiological adaptation process that encompasses various osmoregulatory organs and tissues, including the gills, hepatopancreas, and hemocytes, along with multiple immune and metabolic pathways. Among the DEGs under acute and chronic hypotonic stress, the hemocytes of *P. monodon* were predominantly involved in regulating osmotic pressure, followed by the gills and hepatopancreas, with temporal variations observed among these three organs. The outcomes of GO and KEGG enrichment analyses indicated that the osmoregulatory capacity of *P. monodon* under hypotonic stress was ultimately enhanced due to the involvement of lipids and amino acids in energy metabolism and cell membrane regulation. Consequently, the osmotic regulation of *P. monodon* is primarily controlled by pathways such as lipid metabolism and amino acid metabolism. Furthermore, numerous other DEGs and KEGG pathways may directly or indirectly participate in the osmotic regulation of *P. monodon*. The findings also indicated that osmotic regulation correlates with immune responses, as evidenced by redox processes, activation of cell surface receptor signaling pathways activated by innate immune responses, immune response regulatory signaling pathways, and other immune-related biological processes in hemocytes. In summary, transcriptome analysis reveals the osmotic regulatory mechanisms of *P. monodon* under acute and chronic salinity fluctuations.

## Data Availability

The original contributions presented in the study are included in the article, further inquiries can be directed to the corresponding authors. Raw sequencing data were deposited at NCBI with BioProject accession number PRJNA1137583.

## Footnotes

^1^ https://www.ncbi.nlm.nih.gov/tools/primer-blast/index.cgi
