# Supplementary material for: Transcriptomic analysis of Penaeus monodon in response to acute and chronic hypotonic stress
Source: Front Vet Sci. 2024 Aug 21;11:1464291. doi: 10.3389/fvets.2024.1464291 (PMC11371775; doi:10.3389/fvets.2024.1464291)
Supplement: Supplementary file 1 [file Data_Sheet_1.docx]

Supplementary Material

Table S1 Statistics after quality control

| Sample | Clean reads | Clean bases | Q20 (%) | Q30 (%) | GC content (%) |
| --- | --- | --- | --- | --- | --- |
| SC_Gil_1 | 51657186 | 7723904068 | 98.27 | 94.75 | 46.72 |
| SC_Gil_2 | 56234360 | 8388616629 | 98.26 | 94.72 | 48.04 |
| SC_Gil_3 | 58930750 | 8794835696 | 98.35 | 94.97 | 47.08 |
| SC_Hea_1 | 58828634 | 8792988062 | 97.73 | 93.60 | 47.39 |
| SC_Hea_2 | 57270900 | 8550163257 | 97.70 | 93.51 | 46.33 |
| SC_Hea_3 | 52865856 | 7896412318 | 97.69 | 93.49 | 45.85 |
| SC_Hep_1 | 54164428 | 8107958852 | 98.49 | 95.25 | 50.01 |
| SC_Hep_2 | 58131998 | 8690348431 | 98.47 | 95.25 | 50.54 |
| SC_Hep_3 | 54435948 | 8142736182 | 98.38 | 95.03 | 50.61 |
| J6_Gil_1 | 54493700 | 8126913635 | 98.25 | 94.69 | 46.98 |
| J6_Gil_2 | 54650562 | 8157244909 | 98.36 | 94.95 | 47.77 |
| J6_Gil_3 | 58004652 | 8651659222 | 98.31 | 94.88 | 48.03 |
| J6_Hea_1 | 53386810 | 7969407370 | 98.23 | 94.61 | 43.67 |
| J6_Hea_2 | 46753376 | 7005802774 | 98.15 | 94.41 | 44.07 |
| J6_Hea_3 | 44046846 | 6604666876 | 98.27 | 94.76 | 44.73 |
| J6_Hep_1 | 56532784 | 8438962519 | 98.45 | 95.18 | 50.32 |
| J6_Hep_2 | 56053092 | 8373772630 | 98.47 | 95.20 | 49.83 |
| J6_Hep_3 | 50544094 | 7555072720 | 98.49 | 95.29 | 50.55 |
| J48_Gil_1 | 51882582 | 7739974224 | 98.35 | 94.98 | 47.35 |
| J48_Gil_2 | 54402964 | 8117999981 | 98.37 | 95.00 | 47.98 |
| 48_Gil_3 | 53282232 | 7950998041 | 98.32 | 94.85 | 48.02 |
| J48_Hea_1 | 52979548 | 7936558602 | 98.29 | 94.78 | 44.02 |
| J48_Hea_2 | 62066580 | 9277078249 | 98.28 | 94.73 | 46.64 |
| J48_Hea_3 | 46237666 | 6931518779 | 98.12 | 94.38 | 45.53 |
| J48_Hep_1 | 56218786 | 8409665134 | 98.51 | 95.33 | 49.61 |
| J48_Hep_2 | 51256864 | 7674961525 | 98.41 | 95.05 | 49.53 |
| J48_Hep_3 | 49772626 | 7453765855 | 98.42 | 95.08 | 49.24 |
| J72_Gil_1 | 49724080 | 7444560342 | 98.24 | 94.57 | 45.92 |
| J72_Gil_2 | 52570954 | 7841265962 | 98.23 | 94.63 | 47.33 |
| J72_Gil_3 | 62319000 | 9288207203 | 98.32 | 94.87 | 48.19 |
| J72_Hea_1 | 50623784 | 7554082843 | 98.22 | 94.54 | 44.90 |
| J72_Hea_2 | 52157558 | 7803656938 | 98.29 | 94.76 | 45.53 |
| J72_Hea_3 | 44978016 | 6744652646 | 98.25 | 94.66 | 44.78 |
| J72_Hep_1 | 55521438 | 8294373773 | 98.30 | 94.74 | 48.94 |
| J72_Hep_2 | 57575822 | 8615472549 | 98.46 | 95.19 | 50.50 |
| J72_Hep_3 | 67724128 | 10122372944 | 98.43 | 95.11 | 49.56 |
| M2_Gil_1 | 60099680 | 8956980929 | 97.85 | 94.23 | 47.42 |
| M2_Gil_2 | 63444440 | 9474157802 | 98.08 | 94.61 | 46.56 |
| M2_Gil_3 | 48081368 | 7188145026 | 97.80 | 94.14 | 47.14 |
| M2_Hea_1 | 47262180 | 7079784017 | 97.86 | 94.09 | 42.28 |
| M2_Hea_2 | 49020748 | 7335170166 | 98.06 | 94.58 | 45.92 |
| M2_Hea_3 | 50701624 | 7581513634 | 97.76 | 93.99 | 44.94 |
| M2_Hep_1 | 56479838 | 8449418805 | 97.95 | 94.32 | 49.85 |
| M2_Hep_2 | 55352088 | 8280383123 | 98.15 | 94.74 | 49.86 |
| M2_Hep_3 | 58665054 | 8792261326 | 98.15 | 94.78 | 49.66 |
| M10_Gil_1 | 51199612 | 7561116846 | 98.32 | 94.95 | 48.14 |
| M10_Gil_2 | 57853642 | 8619271355 | 98.35 | 94.97 | 48.06 |
| M10_Gil_3 | 58798354 | 8766524056 | 98.29 | 94.86 | 48.57 |
| M10_Hea_1 | 60373336 | 9041500095 | 98.28 | 94.76 | 47.01 |
| M10_Hea_2 | 52394554 | 7839346548 | 98.20 | 94.55 | 47.13 |
| M10_Hea_3 | 61171278 | 9124749232 | 98.25 | 94.70 | 47.38 |
| M10_Hep_1 | 53812314 | 8050489214 | 98.41 | 95.13 | 50.27 |
| M10_Hep_2 | 54669228 | 8171746937 | 98.24 | 94.80 | 51.36 |
| M10_Hep_3 | 59650652 | 8921798827 | 98.34 | 94.92 | 50.46 |
| M15_Gil_1 | 55634068 | 8303514455 | 98.26 | 94.71 | 48.29 |
| M15_Gil_2 | 58103482 | 8675323526 | 98.37 | 95.00 | 49.46 |
| M15_Gil_3 | 55798842 | 8329959970 | 98.25 | 94.70 | 47.97 |
| M15_Hea_1 | 55057260 | 8225258849 | 98.18 | 94.53 | 47.58 |
| M15_Hea_2 | 55231340 | 8227749334 | 98.19 | 94.54 | 47.19 |
| M15_Hea_3 | 53354362 | 7982472490 | 98.18 | 94.52 | 46.53 |
| M15_Hep_1 | 54712620 | 8173045834 | 98.44 | 95.21 | 51.92 |
| M15_Hep_2 | 54034776 | 8080005362 | 98.41 | 95.13 | 51.08 |
| M15_Hep_3 | 54874988 | 8169240946 | 98.31 | 94.92 | 50.33 |

Note: J6, J48, J72 indicated 6 h, 48 h, 72 h after acute hypotonic stress, respectively; M2, M10, M15 indicated 2 d, 10 d, 15 d after chronic hypotonic stress, respectively.


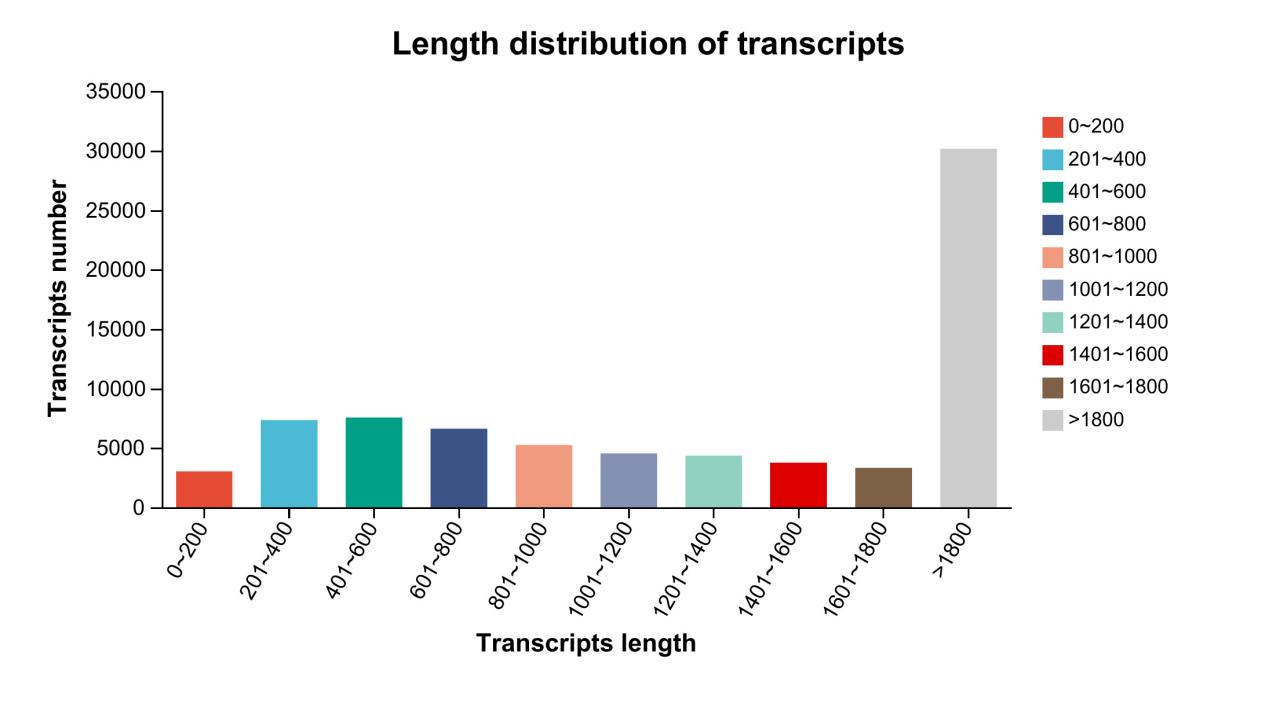


Figure S1. Length distribution of transcripts


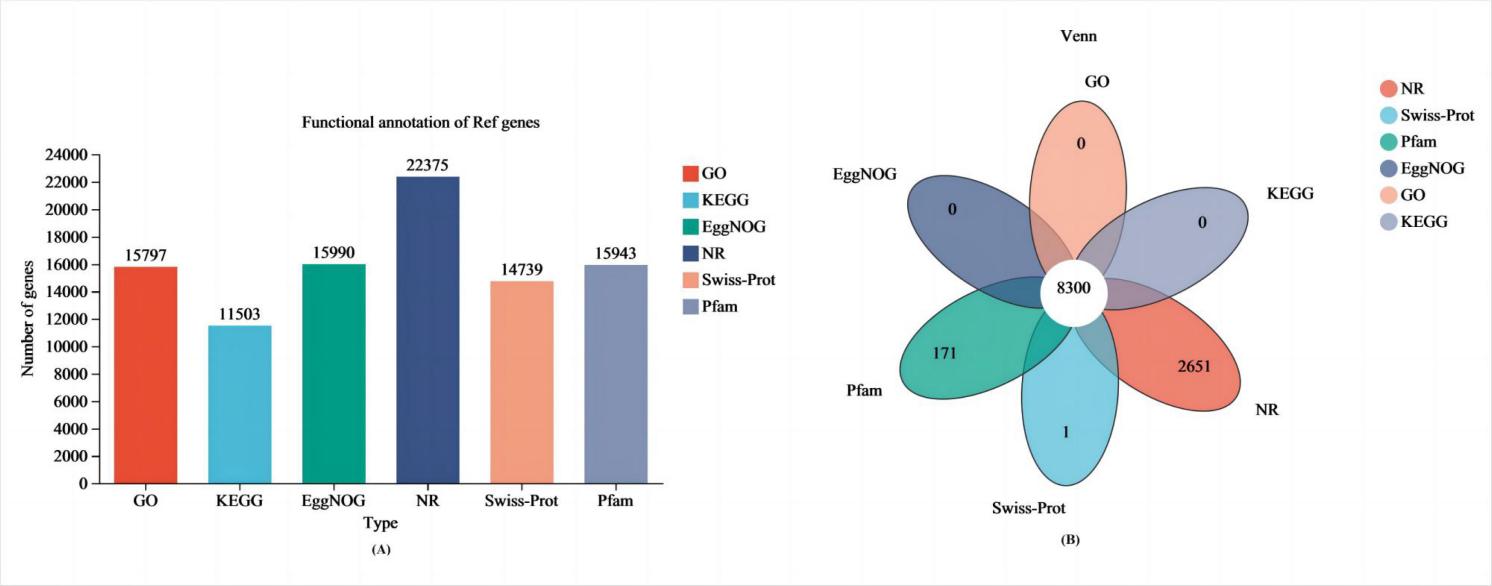


Figure S2. Statistics of functional annotation

Note: A: The horizontal axis represents the database name, and the vertical axis represents the number of sequences annotated to the database; B: The circles of different colors represent the number of genes annotated to different databases, and the intersecting areas of the circles represent the number of genes annotated to multiple databases simultaneously.


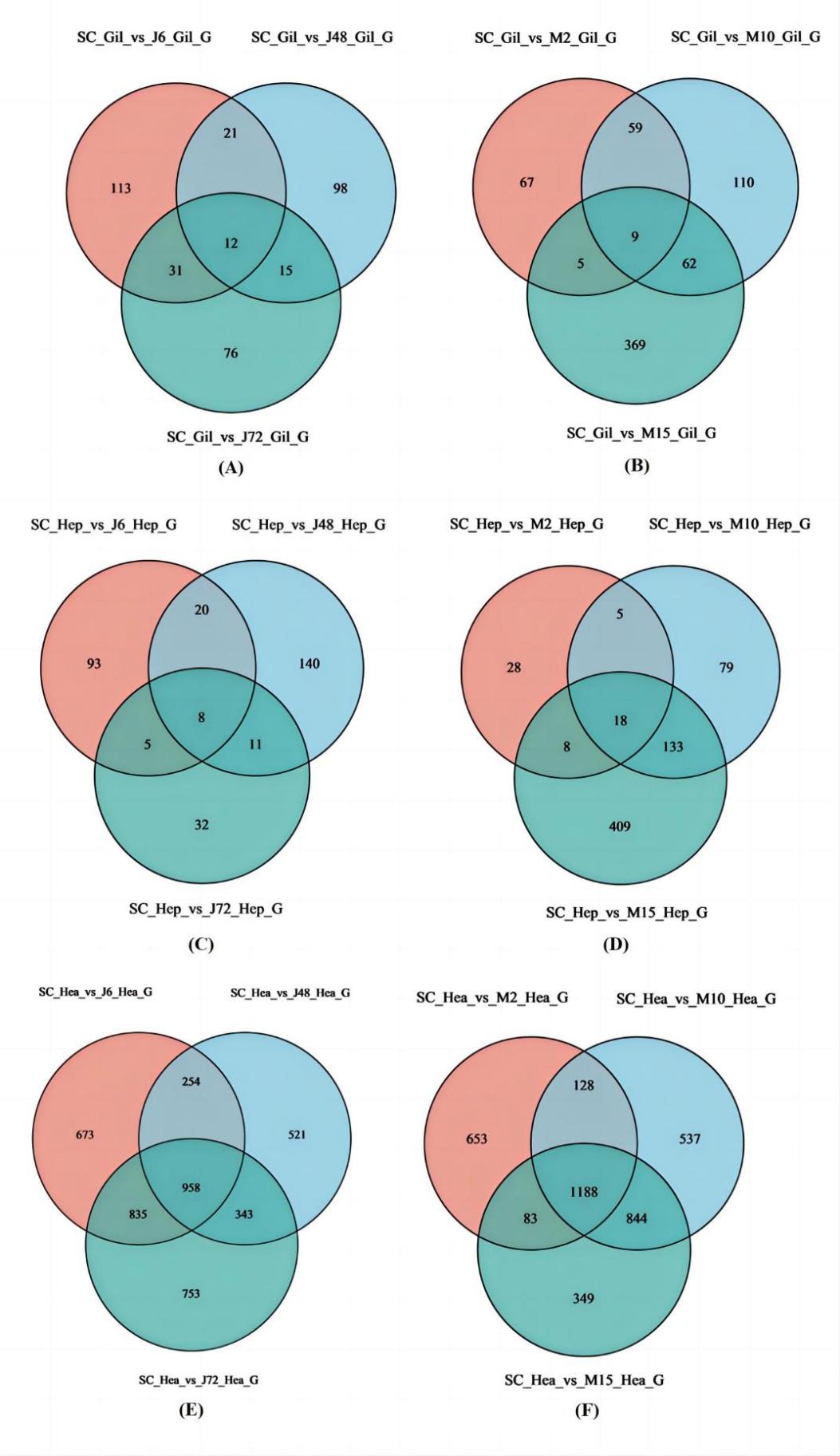


Figure S3. Quantitative statistics of DEGs in gills, hepatopancreas and hemocytes of *P*. *monodon* under acute and chronic hypotonic stress

Note: A and B: gills, C and D: hepatopancreas, E and F: hemocytes.


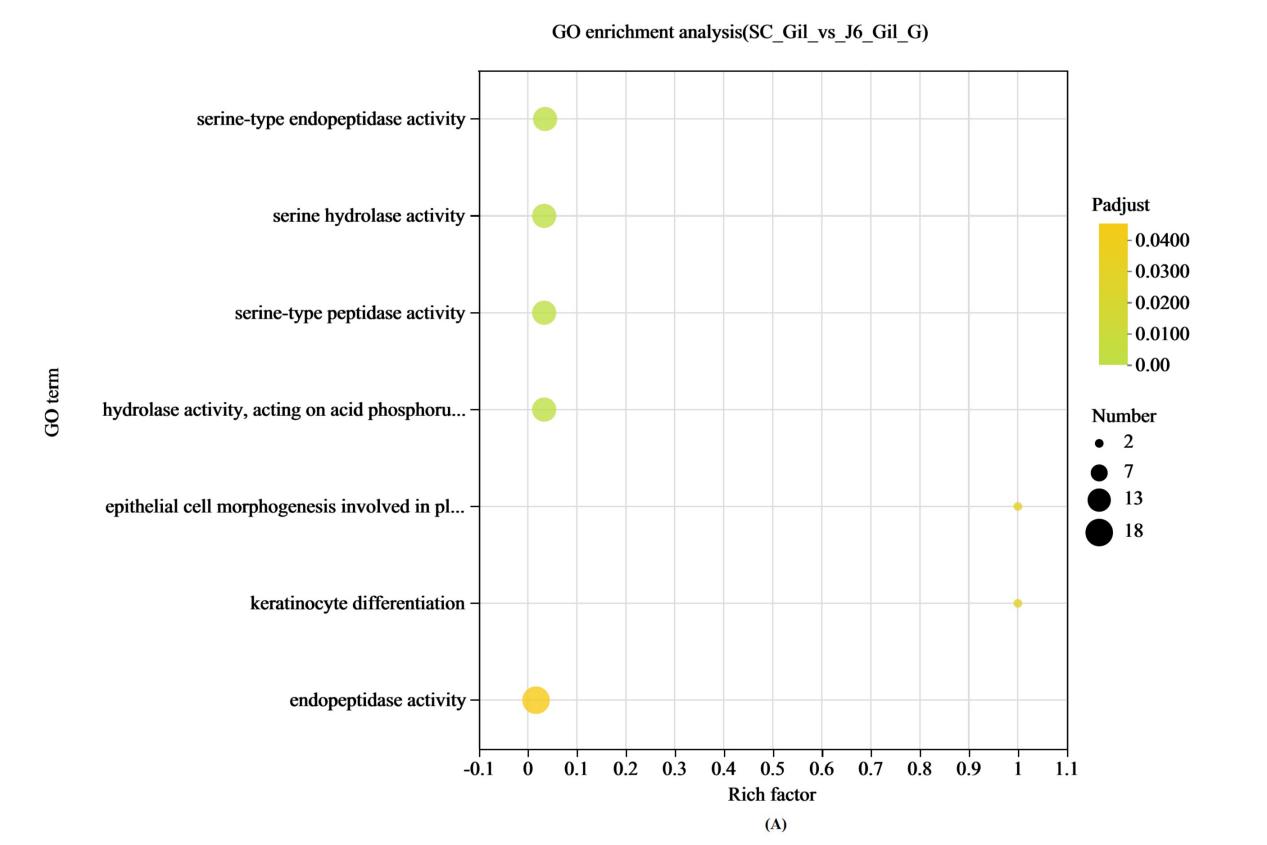


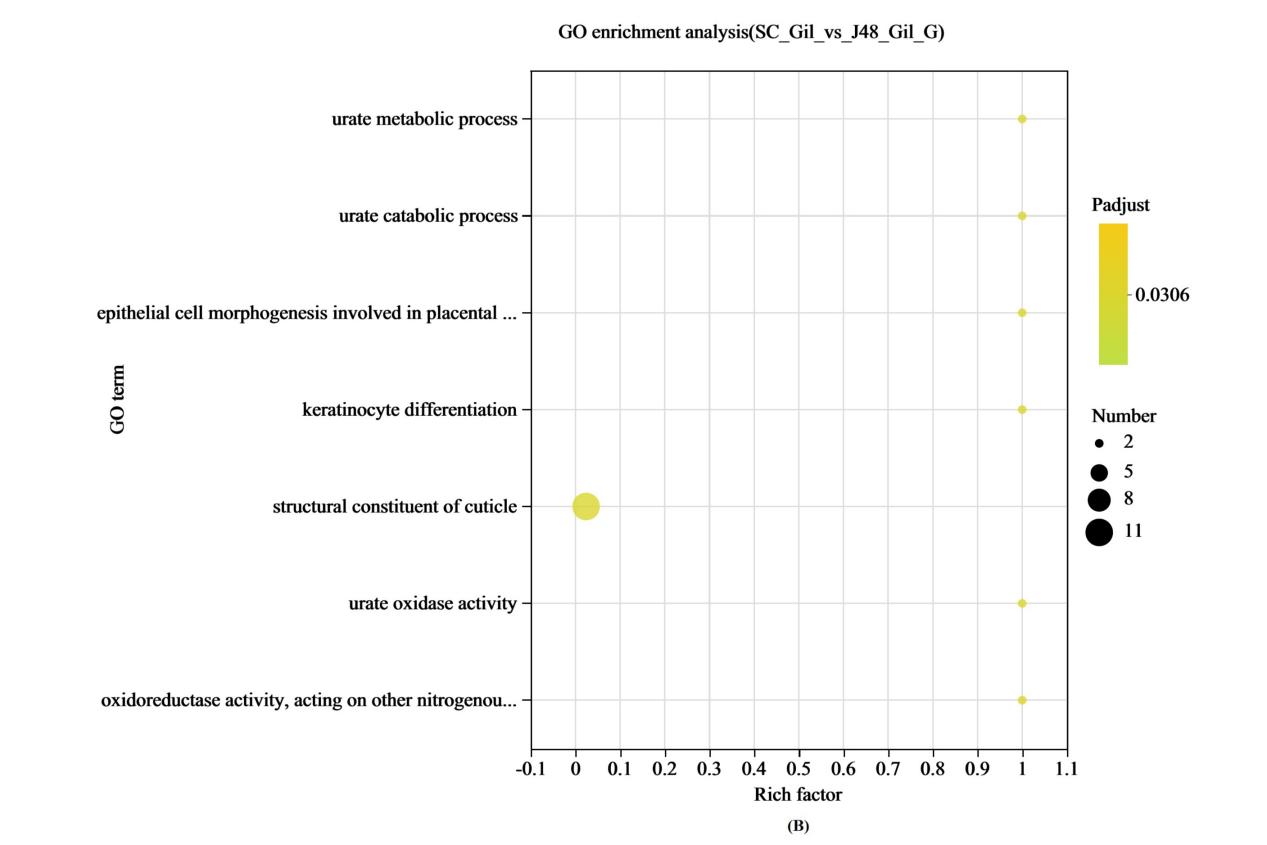


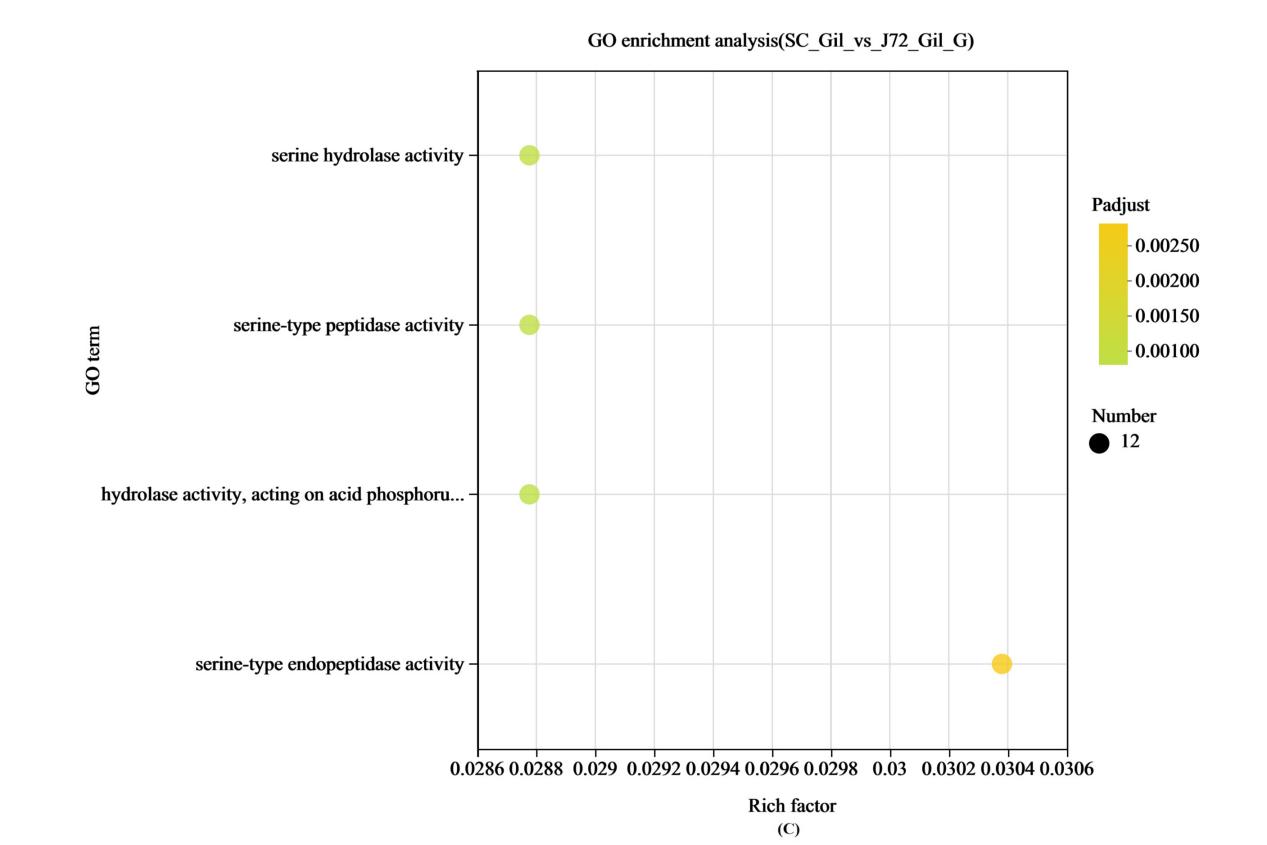


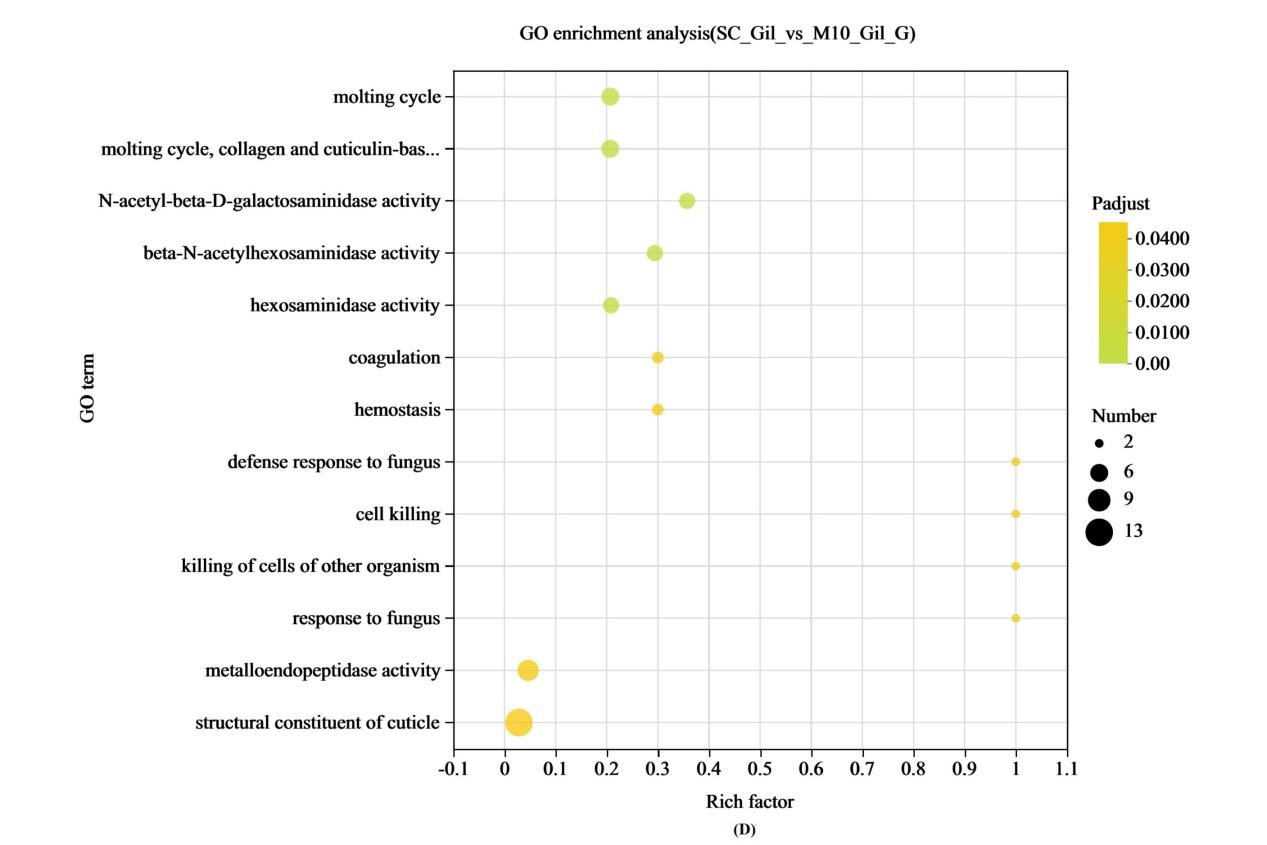


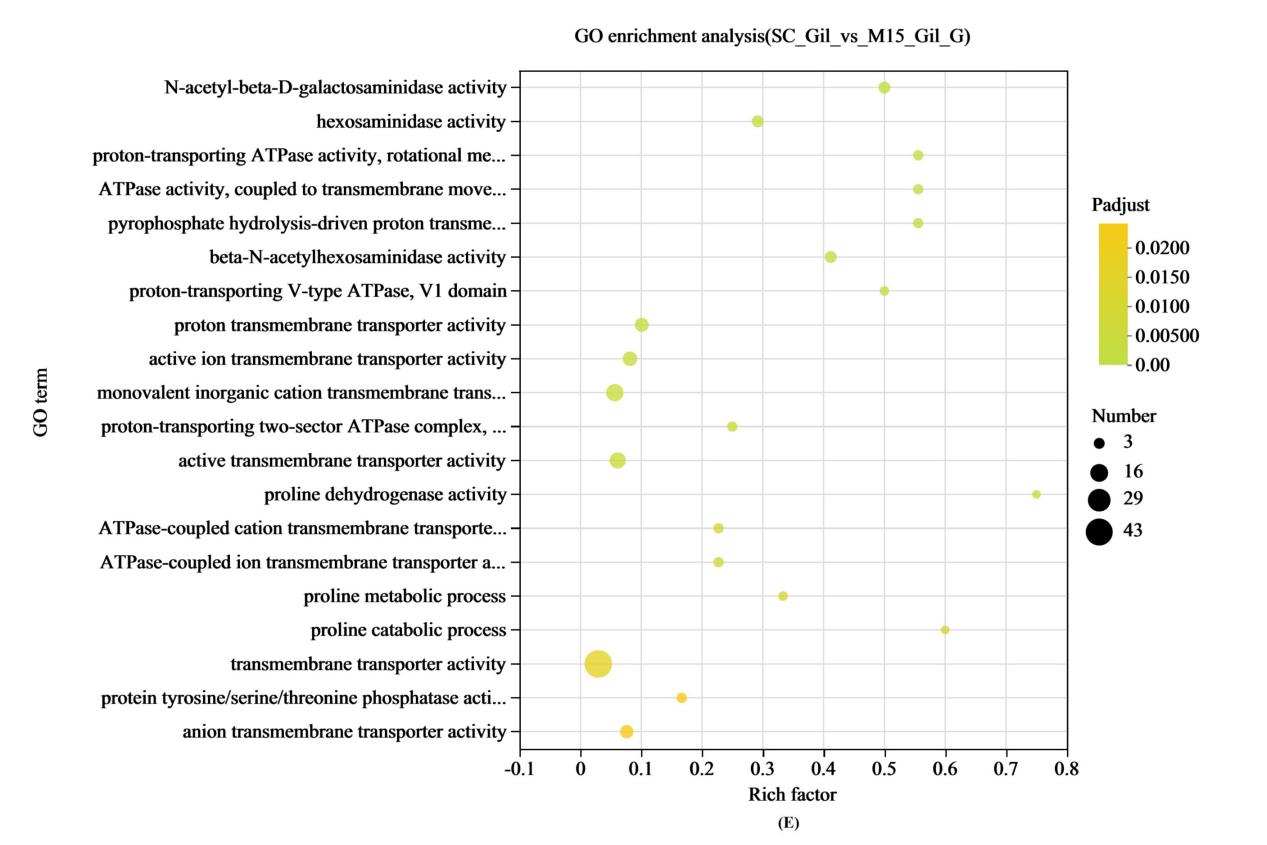


Figure S4. GO enrichment analysis of acute and chronic hypotonic stress in the gills of *P. monodon*

Notes: A, B, C depict the GO enrichment analysis of samples under acute hypotonic stress for 6 h, 48 h, 72 h and the control group. D, E illustrate the GO enrichment analysis comparing the control group with samples under chronic hypotonic stress for 10 d and 15 d.


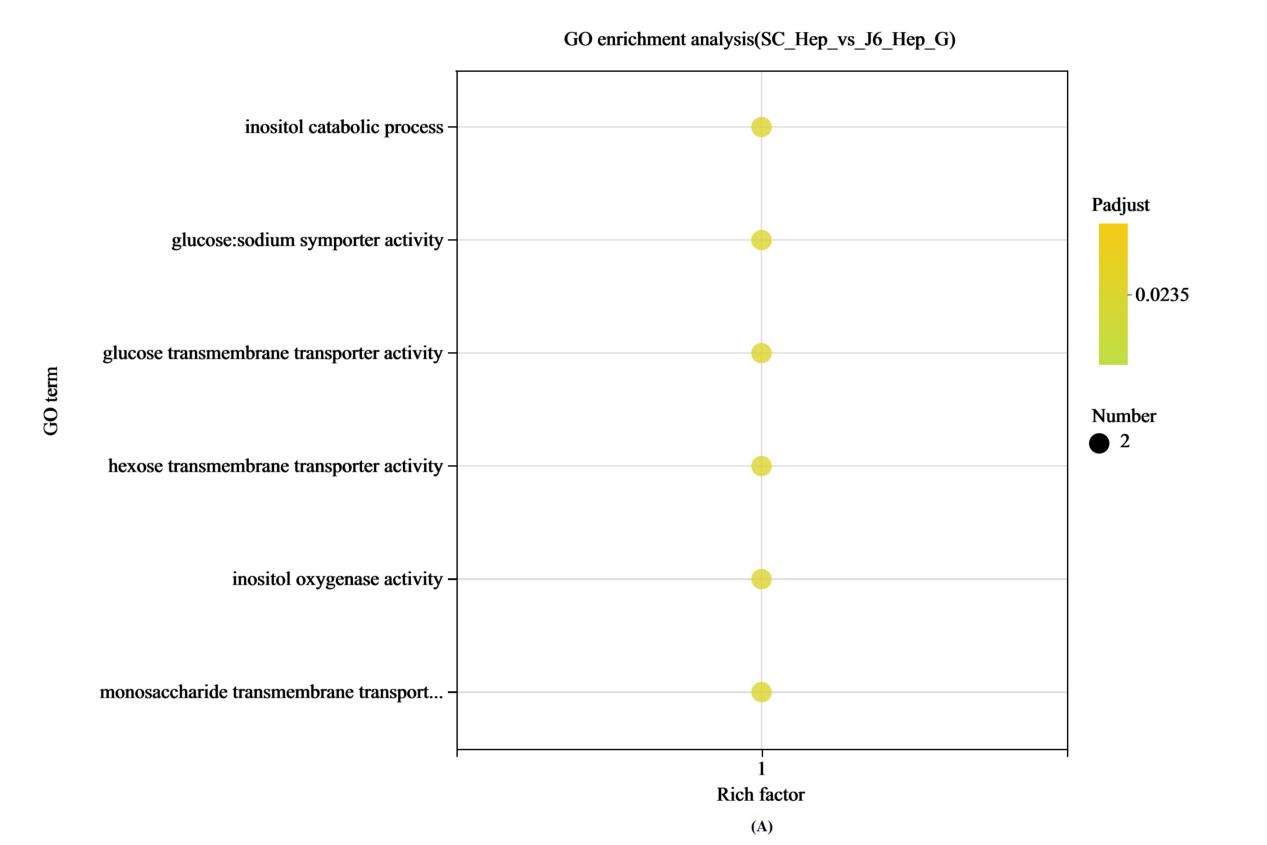


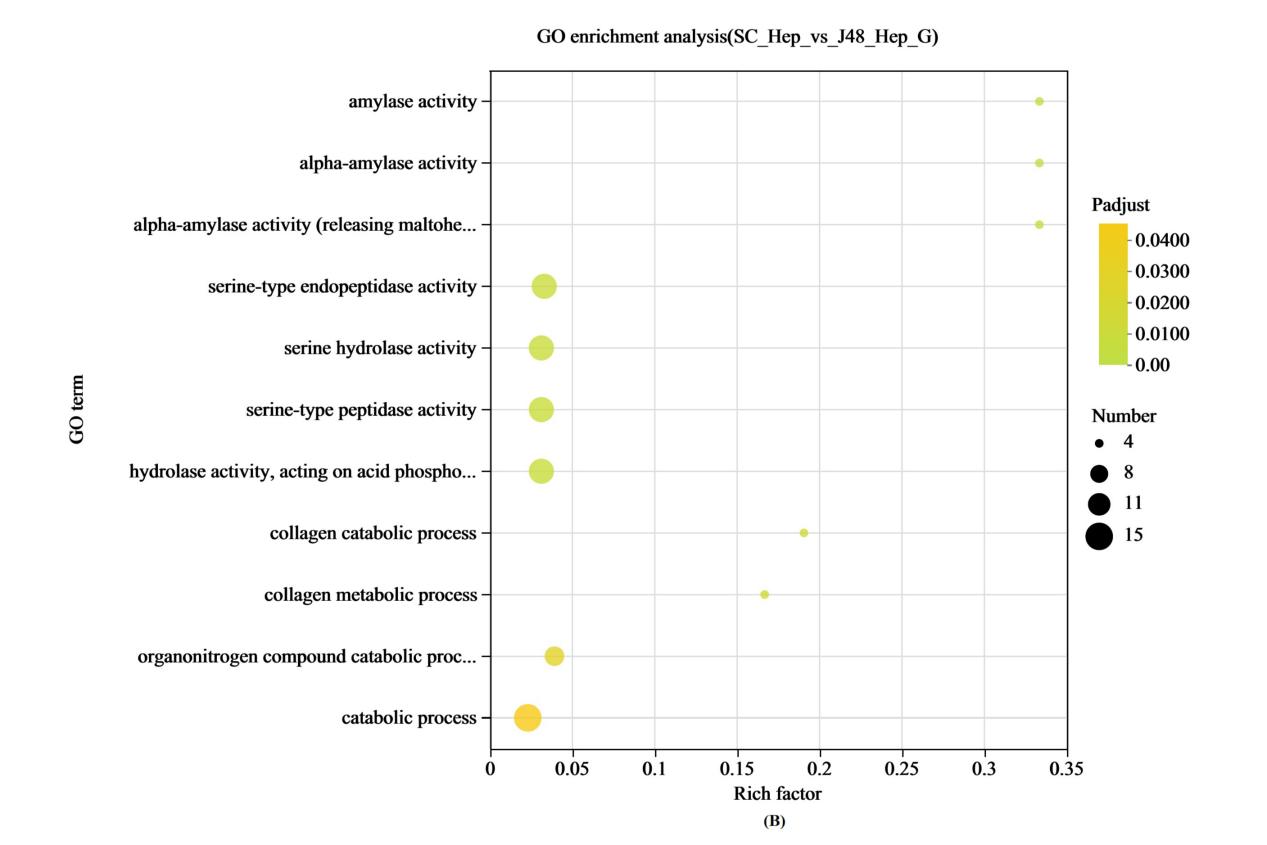

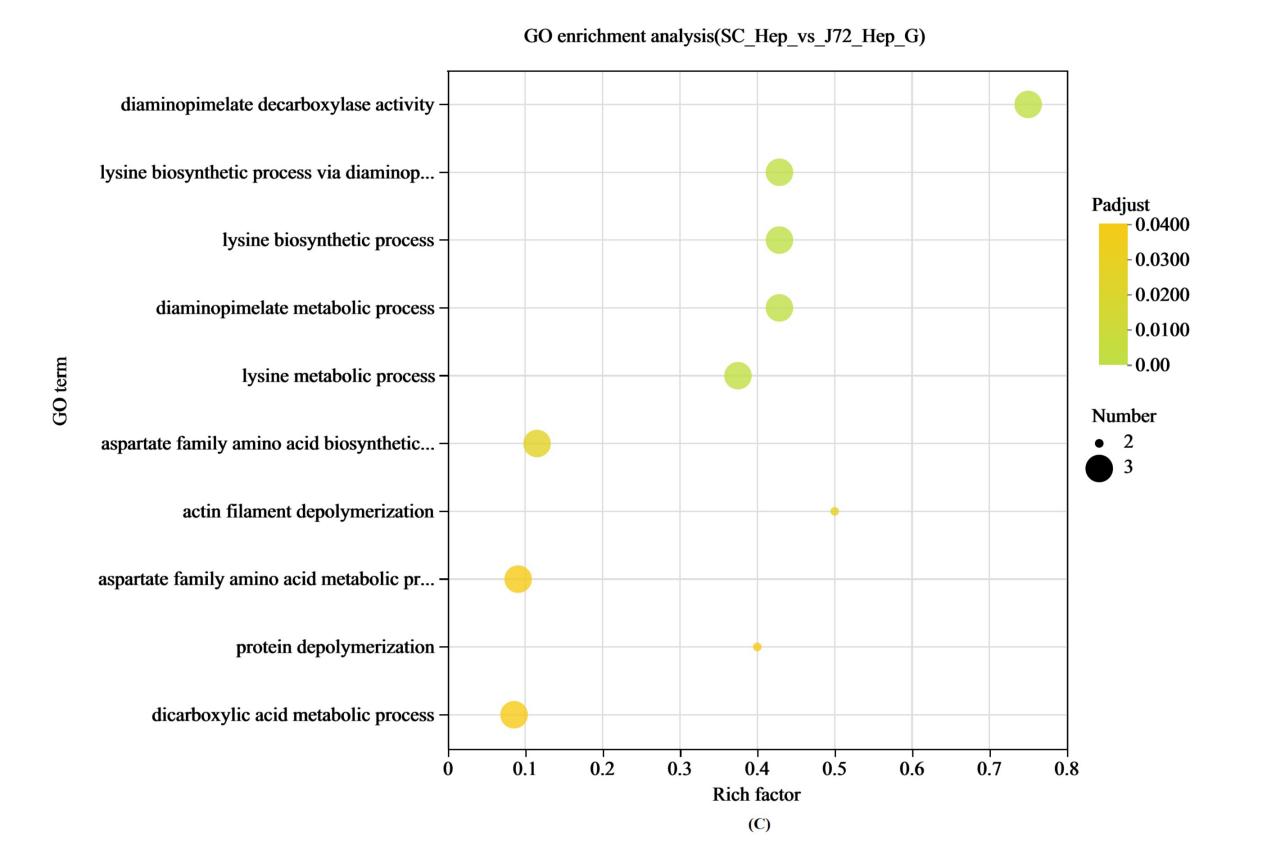

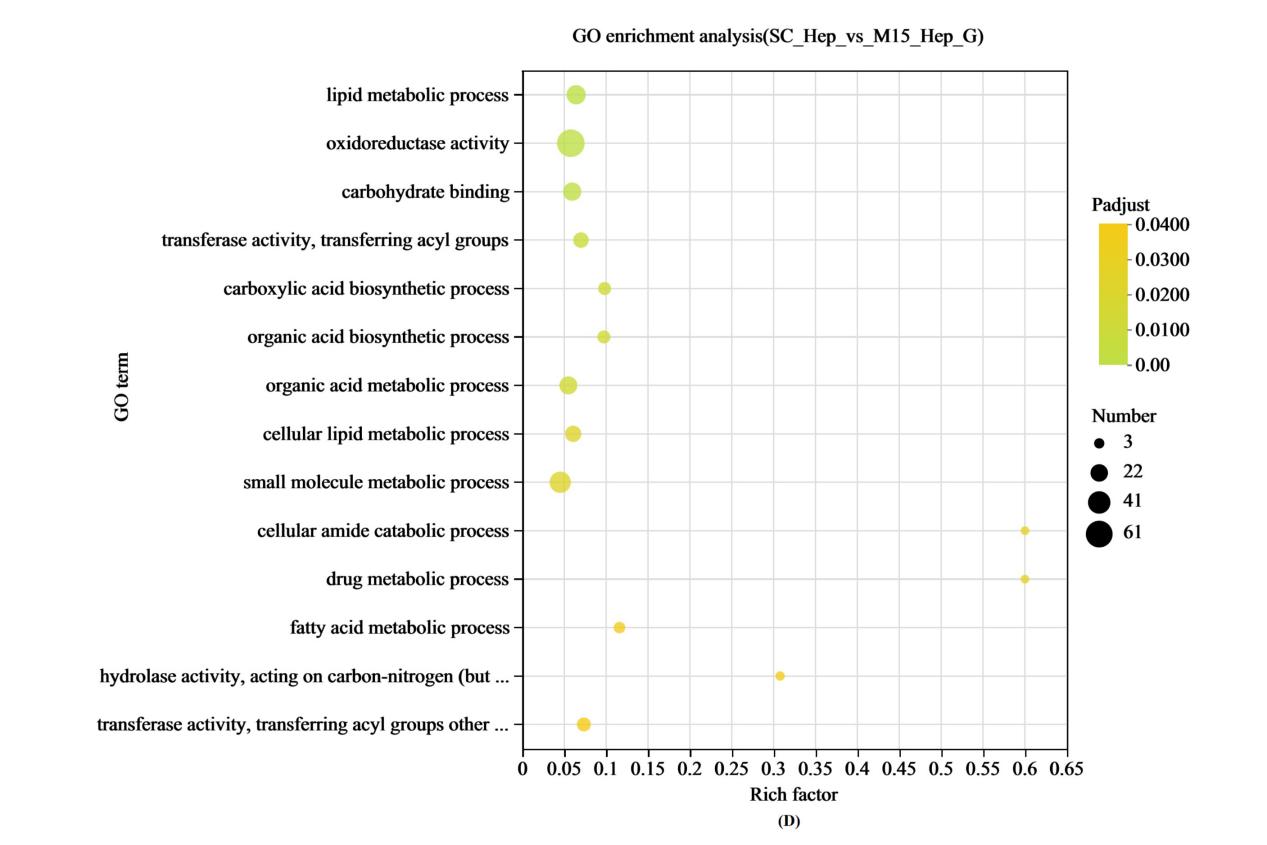


Figure S5. GO enrichment analysis of acute and chronic hypotonic stress in the hepatopancreas of *P. monodon*

Notes: A, B, C display the GO enrichment analysis of samples under acute hypotonic stress for 6 h, 48 h, 72 h and the control group. D shows the GO enrichment analysis comparing the control group with samples under chronic hypotonic for 15 d.


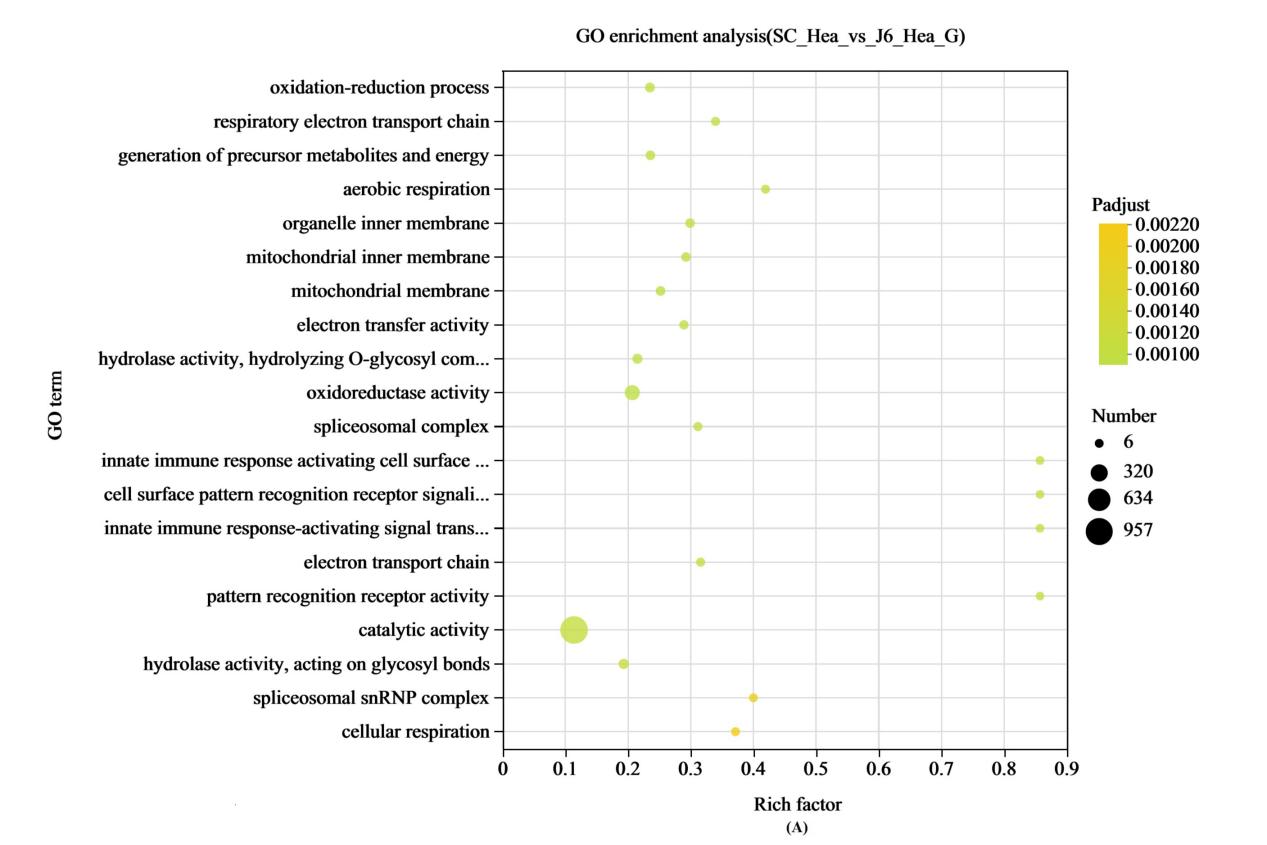

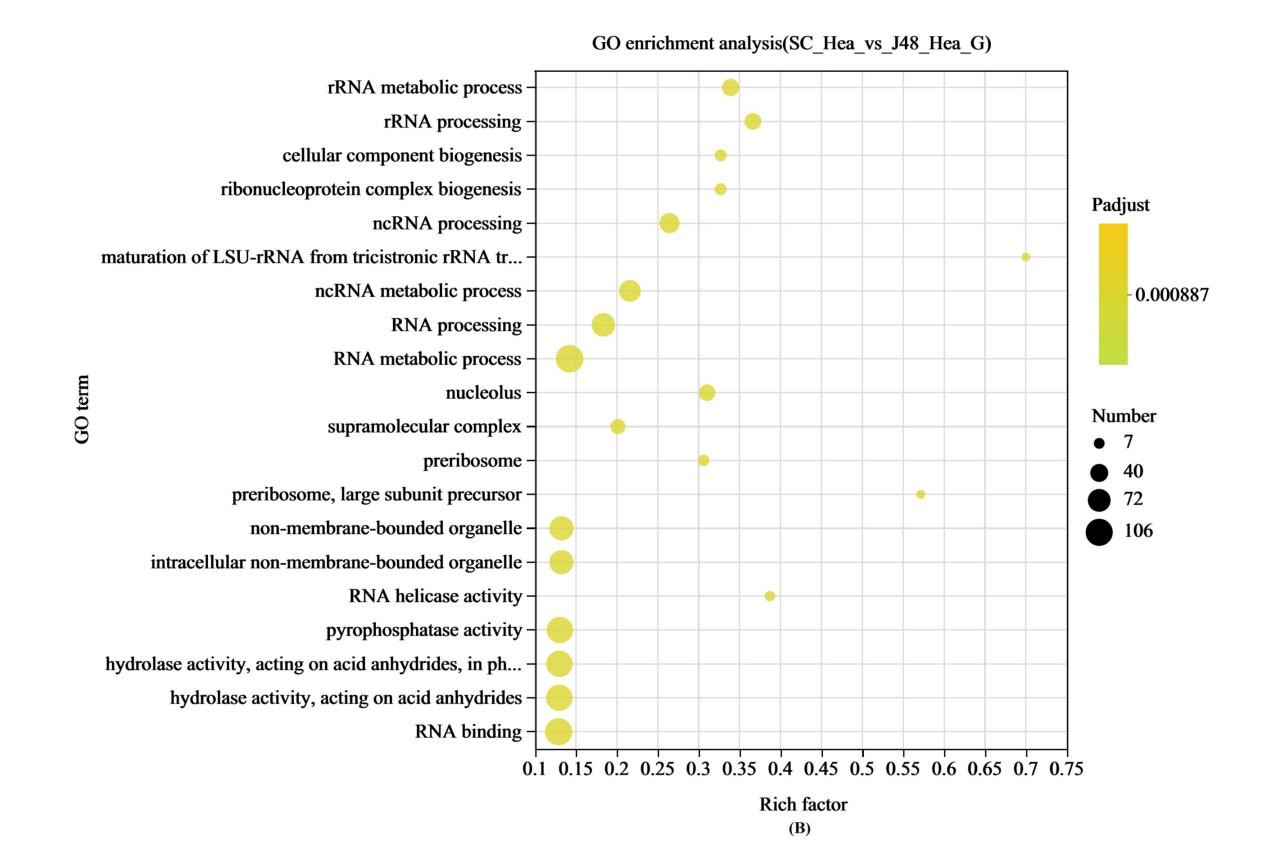


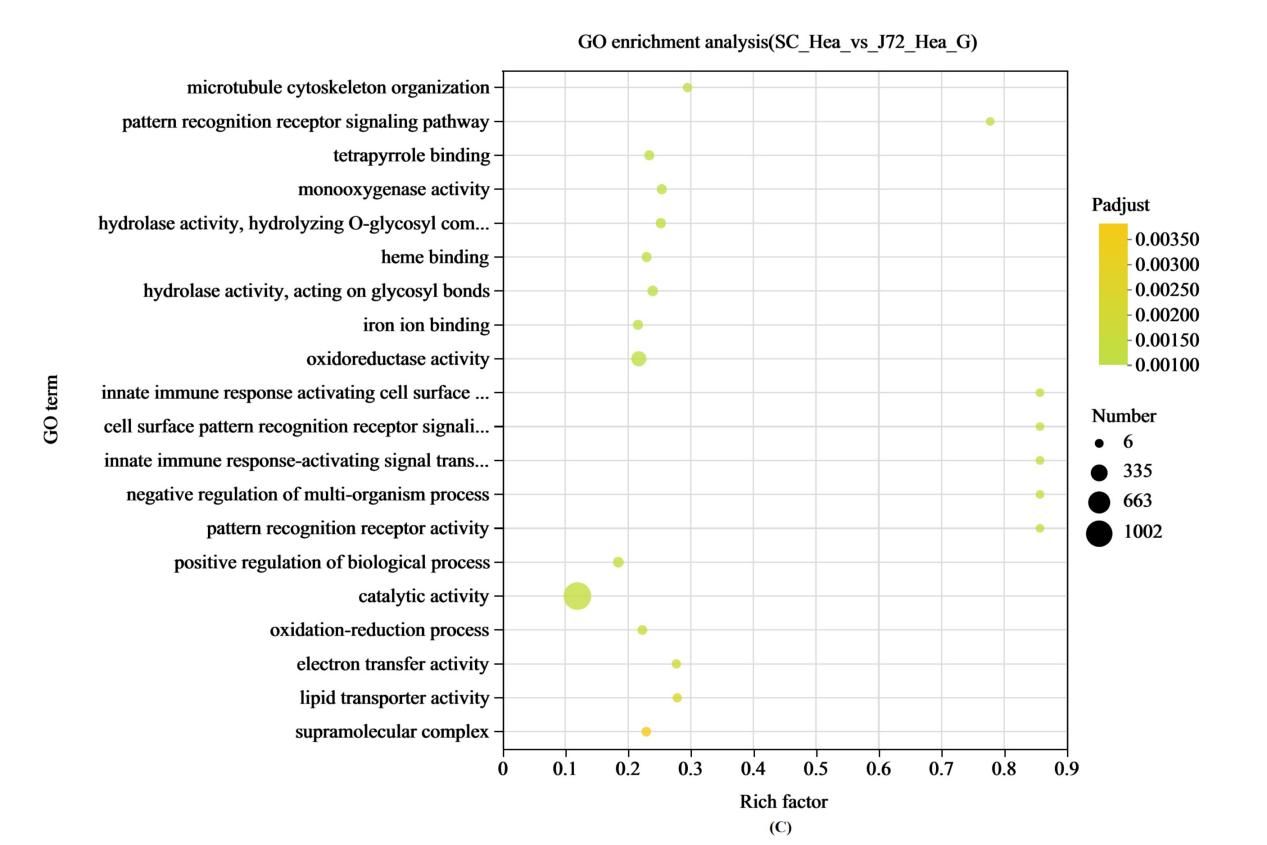


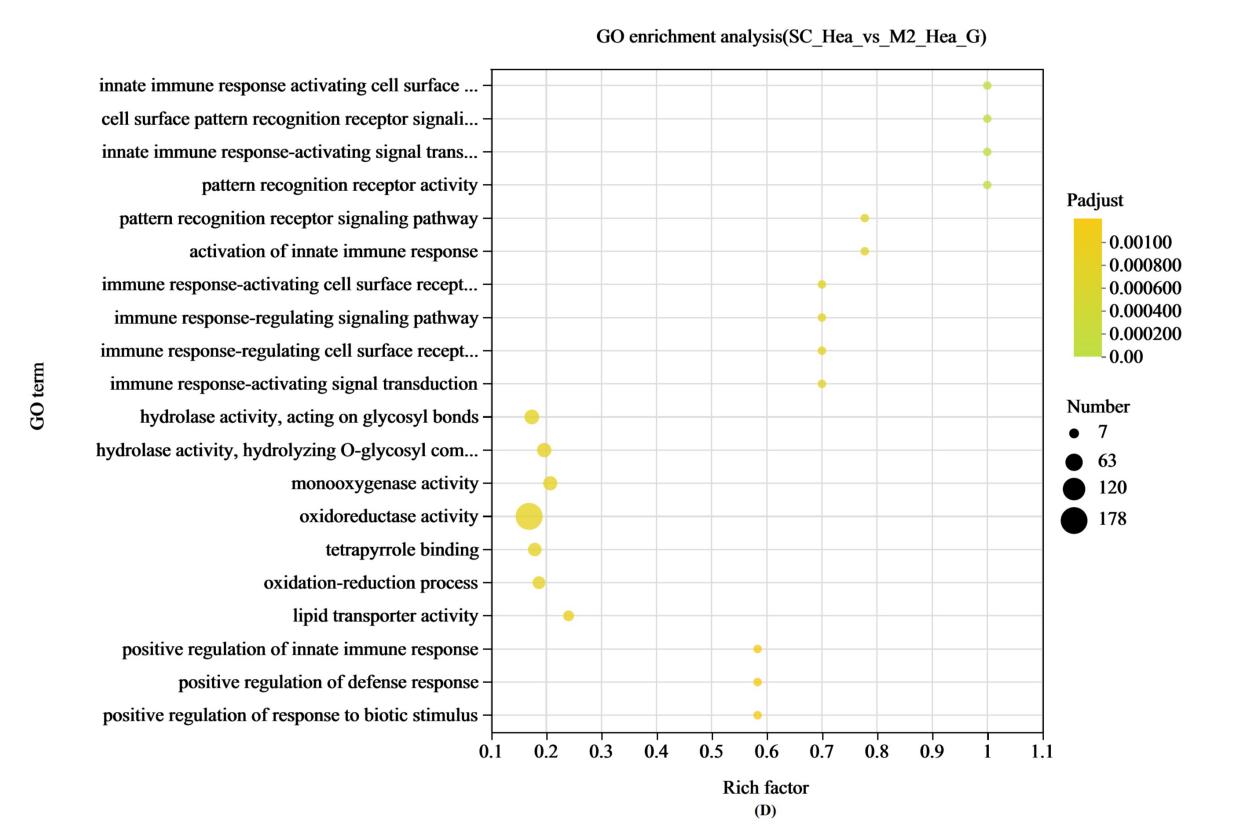


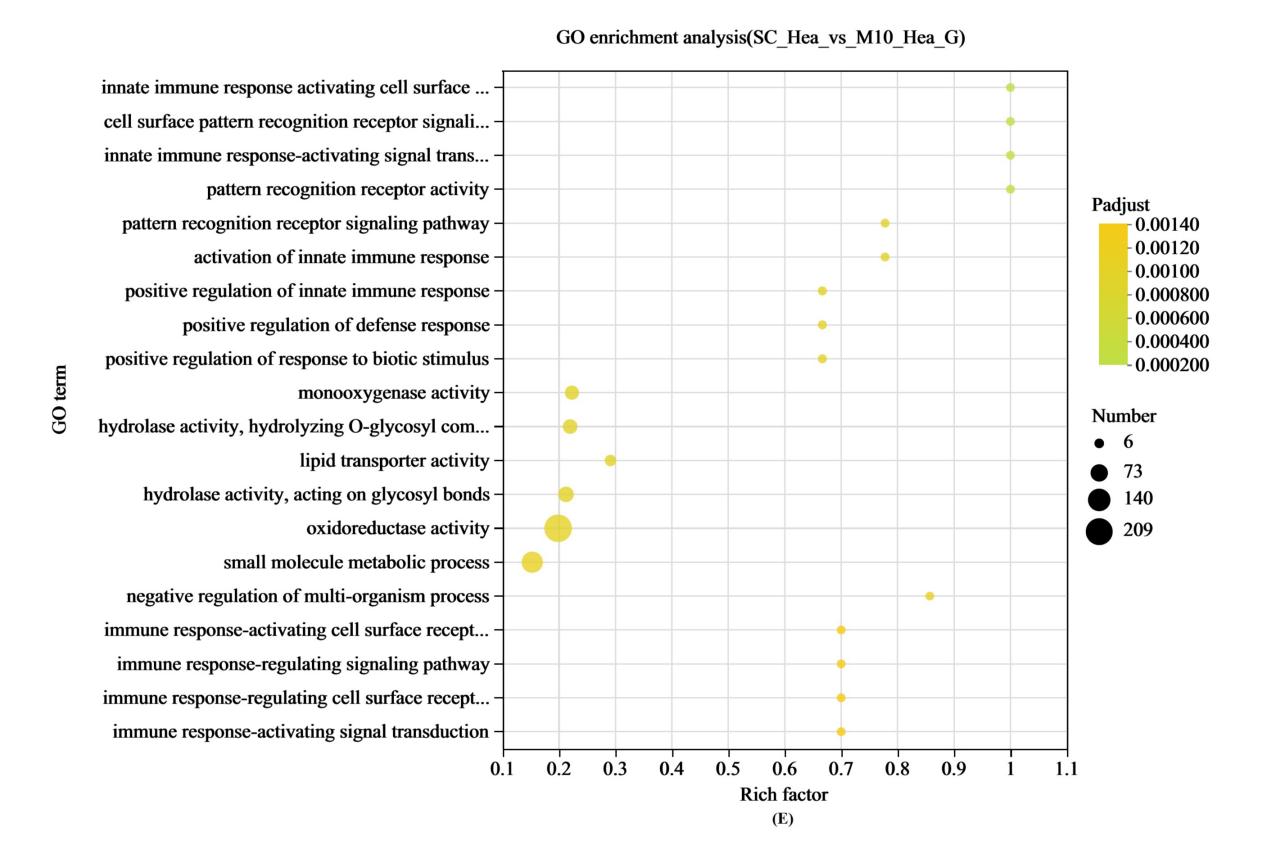

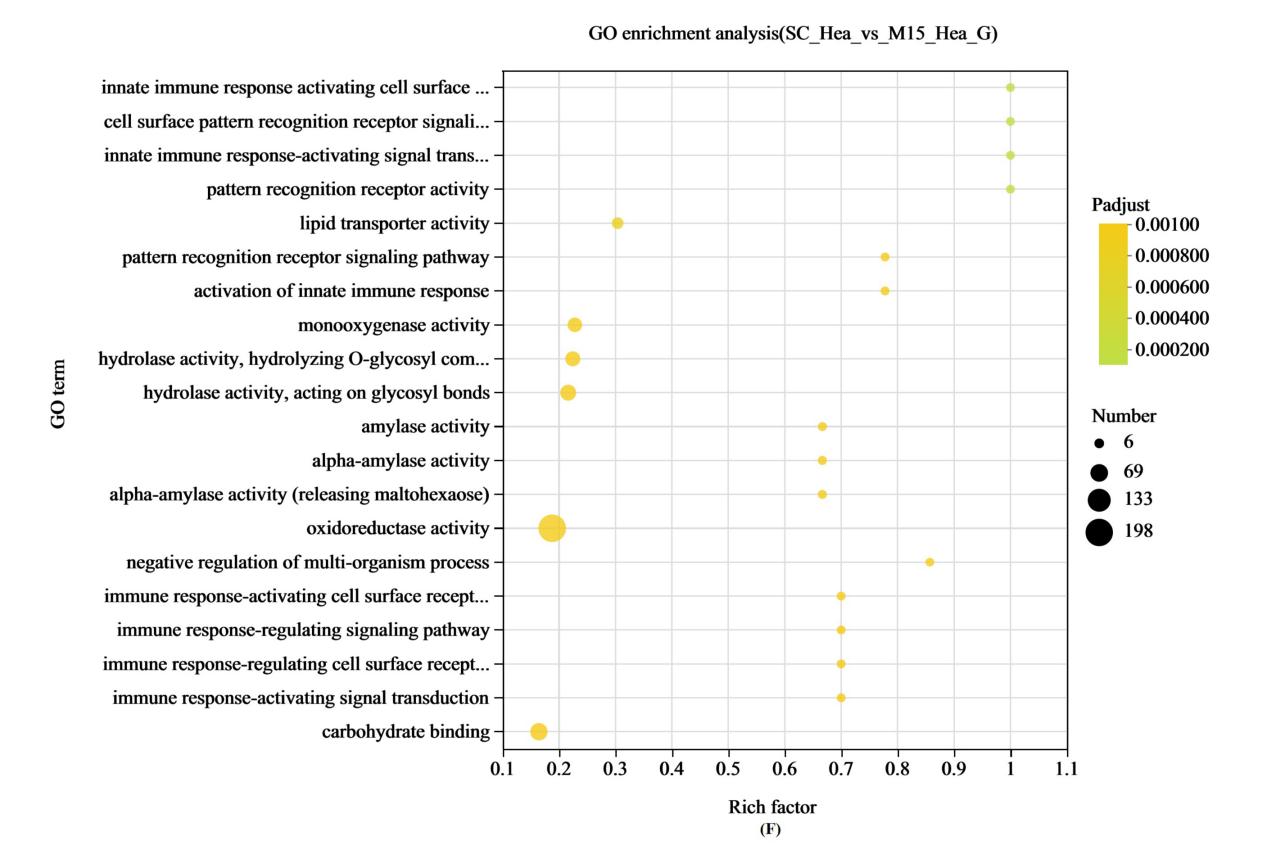


Figure S6. GO enrichment analysis of acute and chronic hypotonic stress in the hemocytes of

*P. monodon*

Notes: A, B, C showed the GO enrichment analysis of samples under acute hypotonic stress for 6 h, 48 h and 72 h with the control group. D, E and F illustrate the GO enrichment analysis comparing the control group with samples under chronic hypotonic stress for 2 d, 10 d and 15 d. The vertical axis represents the GO Term and the horizontal axis represents the Rich factor (the ratio of the number of genes/transcripts enriched to the number of annotated genes in the GO term. A larger Rich factor signifies a higher degree of enrichment). The size of the dots indicates the number of genes in this GO Term, while the colors of the dots correspond to different Padjust ranges.


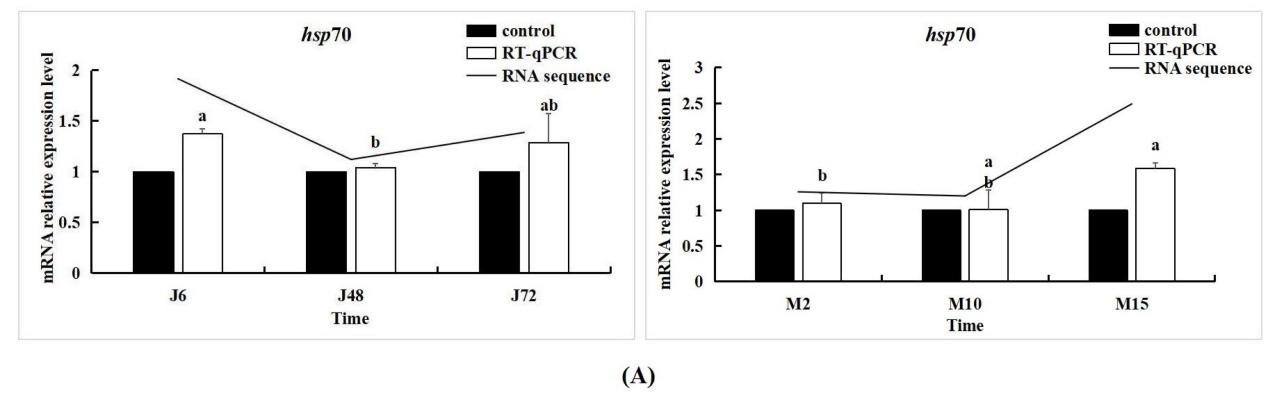


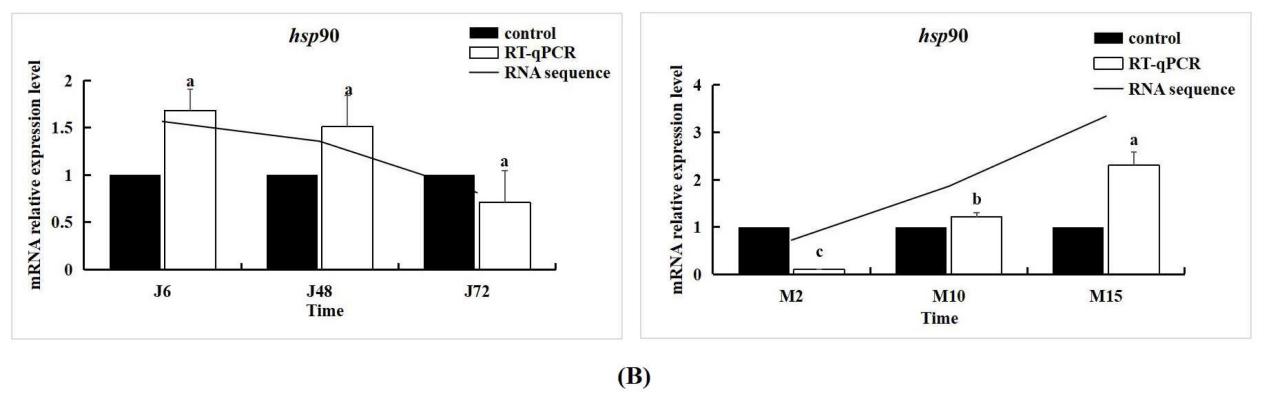


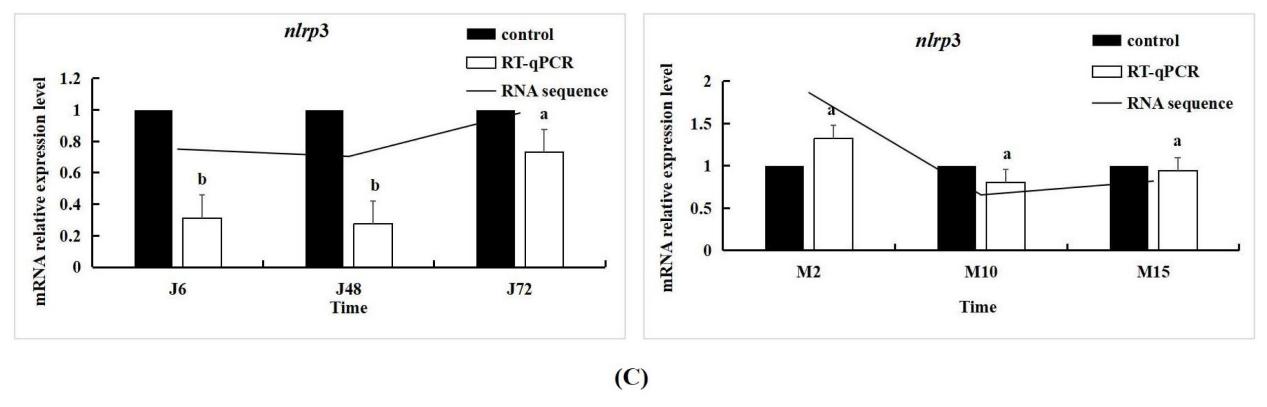


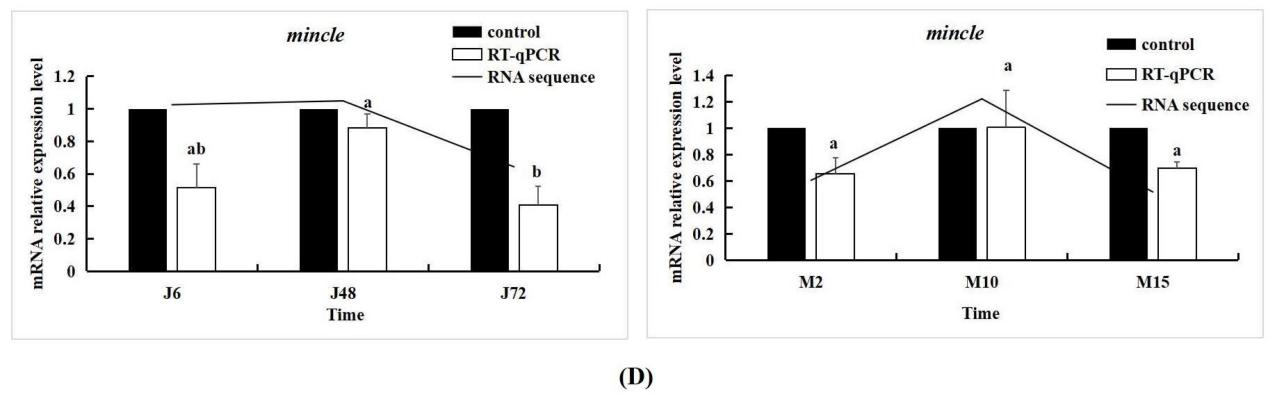


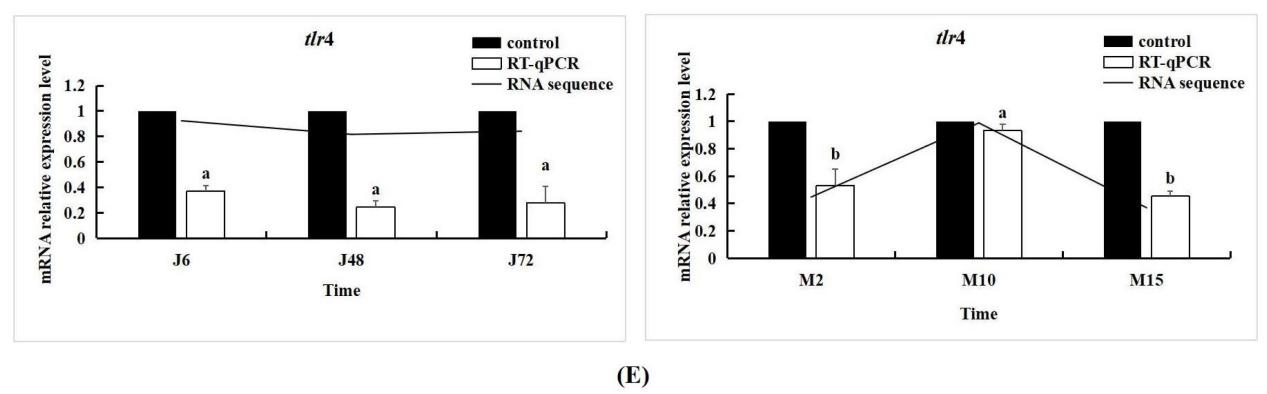


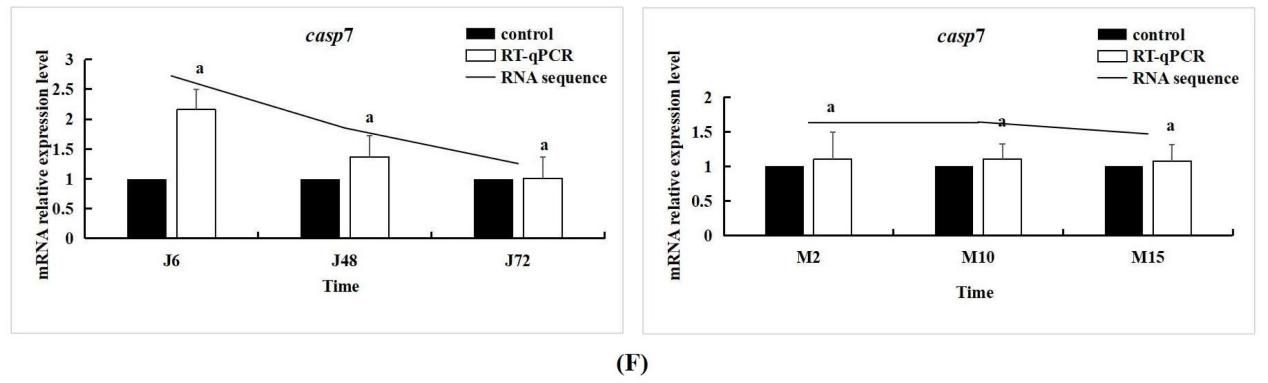


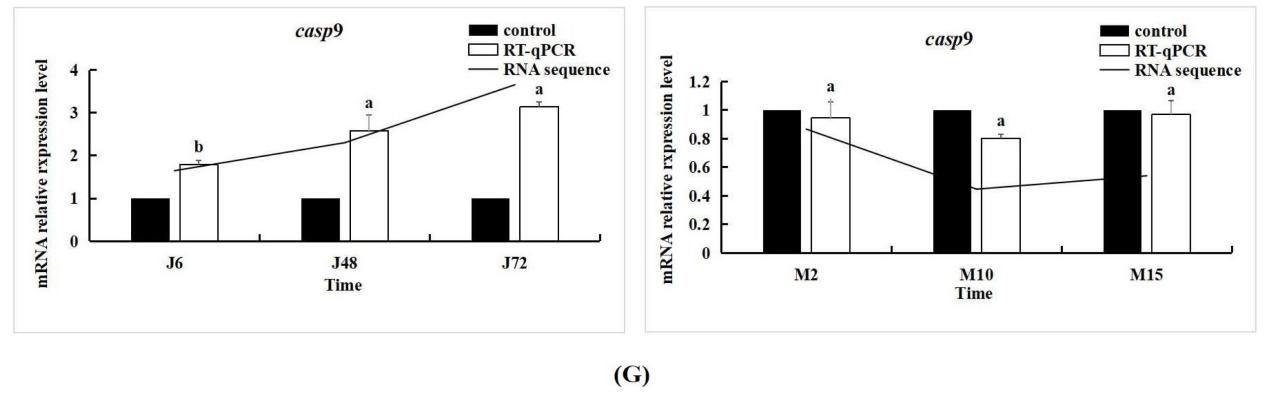


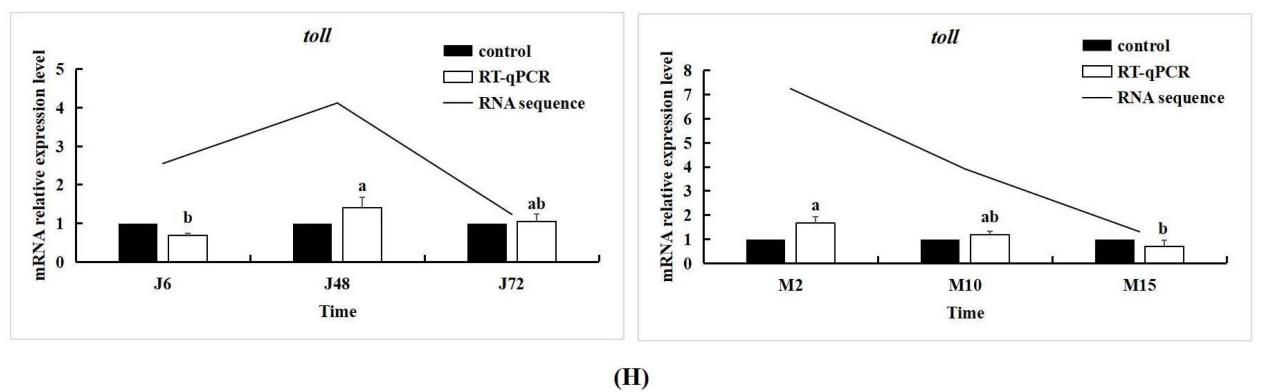


Figure S7. RT-qPCR verification results of DEGs in the gills

Note: A: *hsp*70, B: *hsp*90, C: *nlrp*3, D: *mincle*, E: *trl*4, F: *casp*7, G: *casp*9, H: *toll*."control" indicates the expression of related genes expression under salinity at 20 psu.


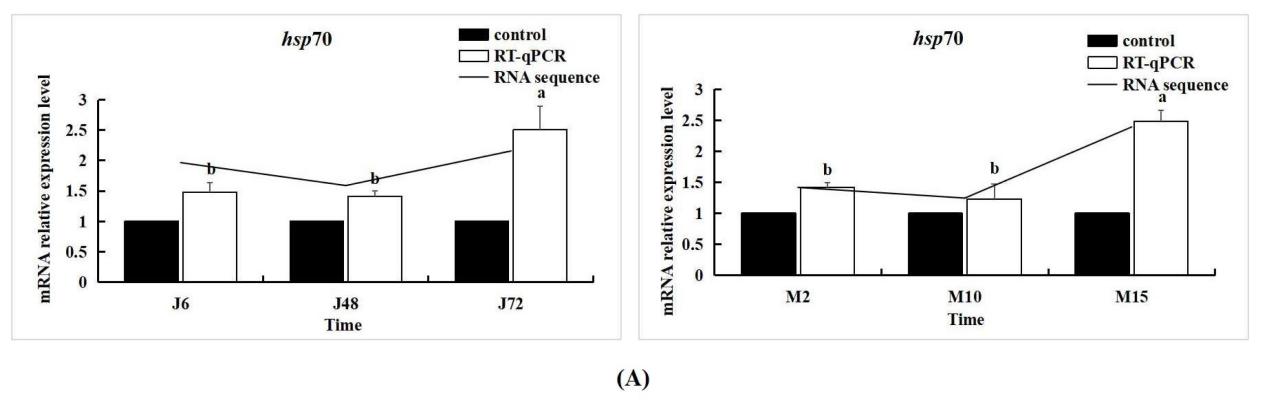

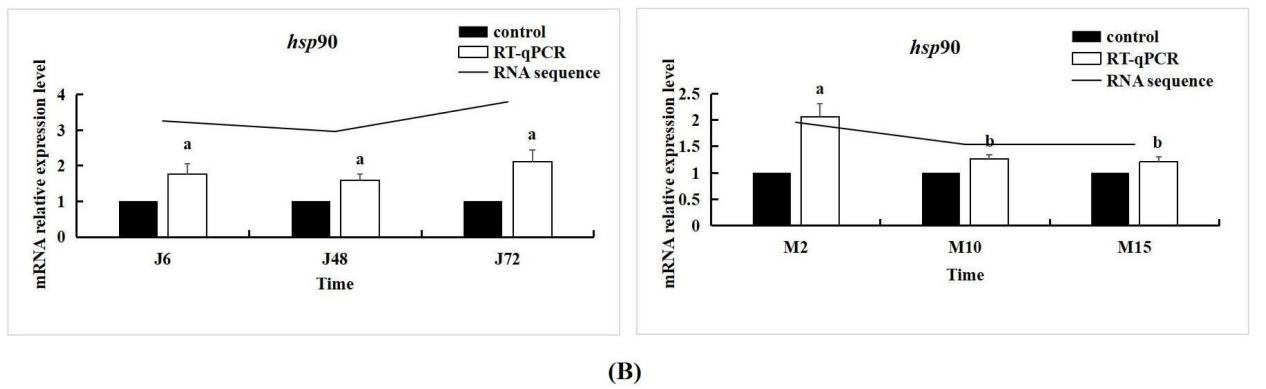

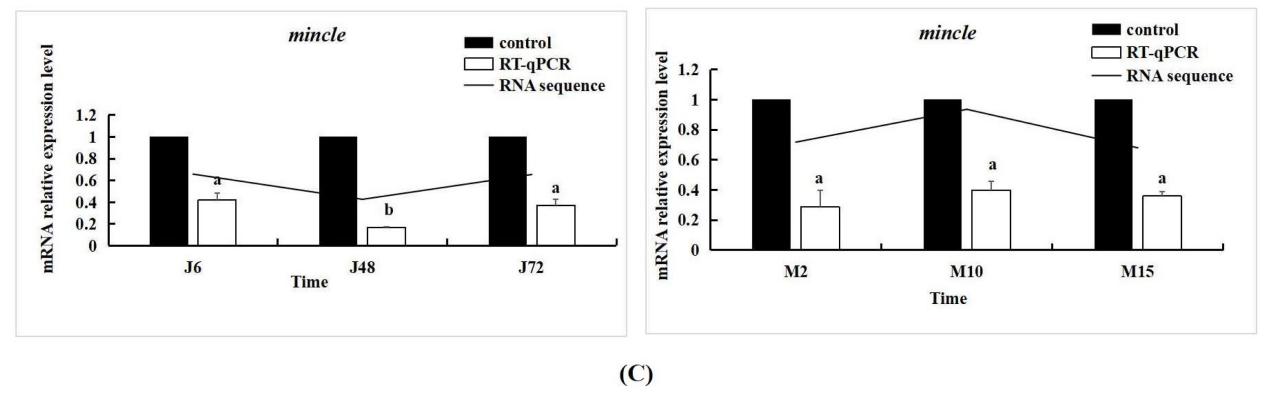

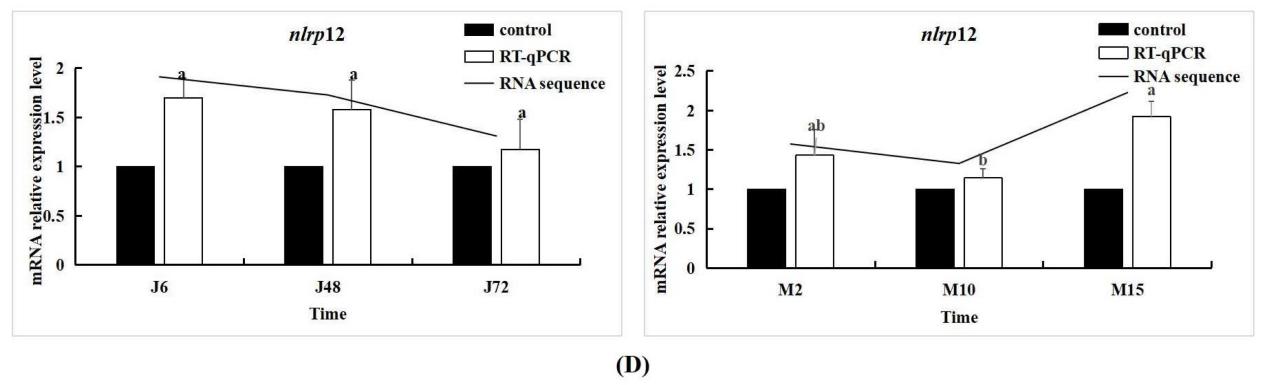

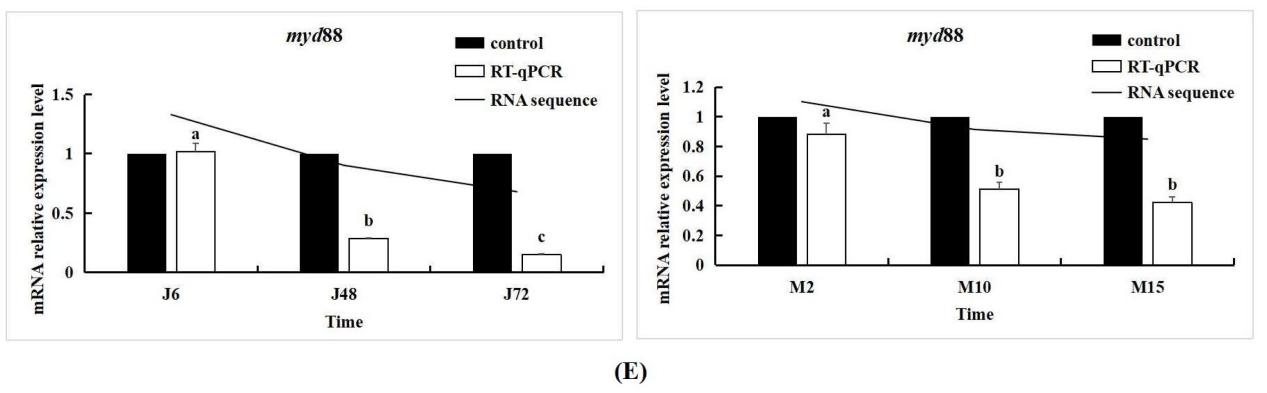

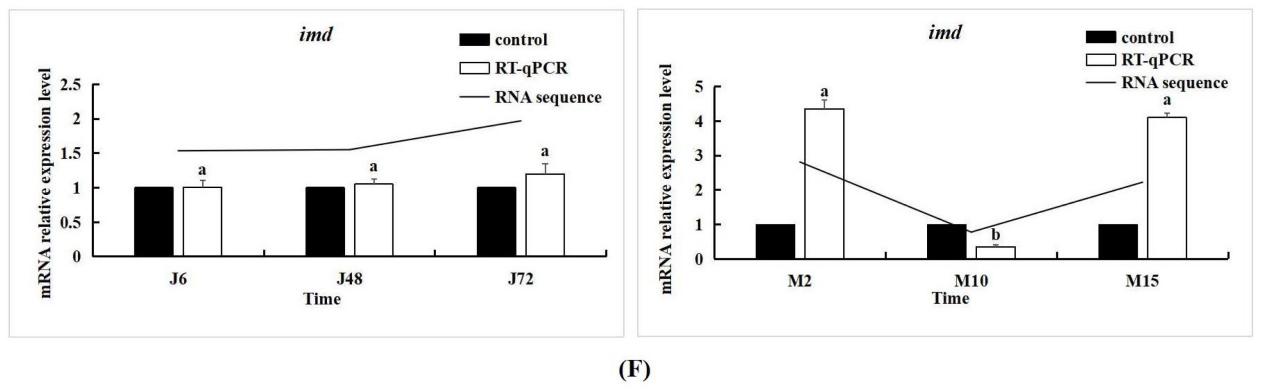

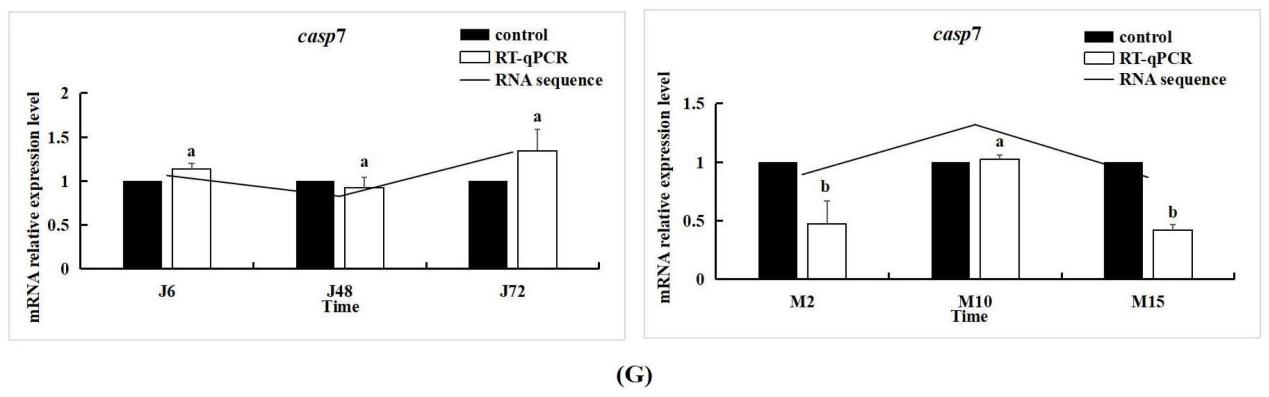

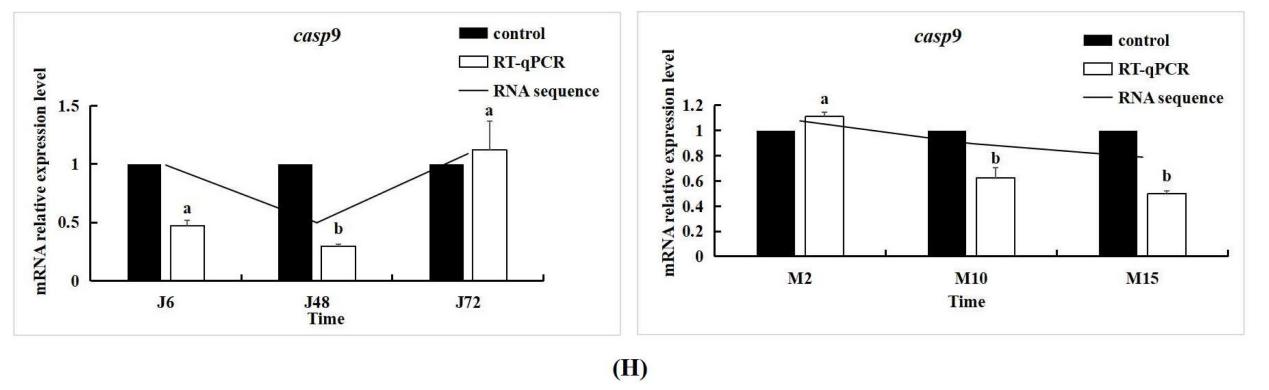

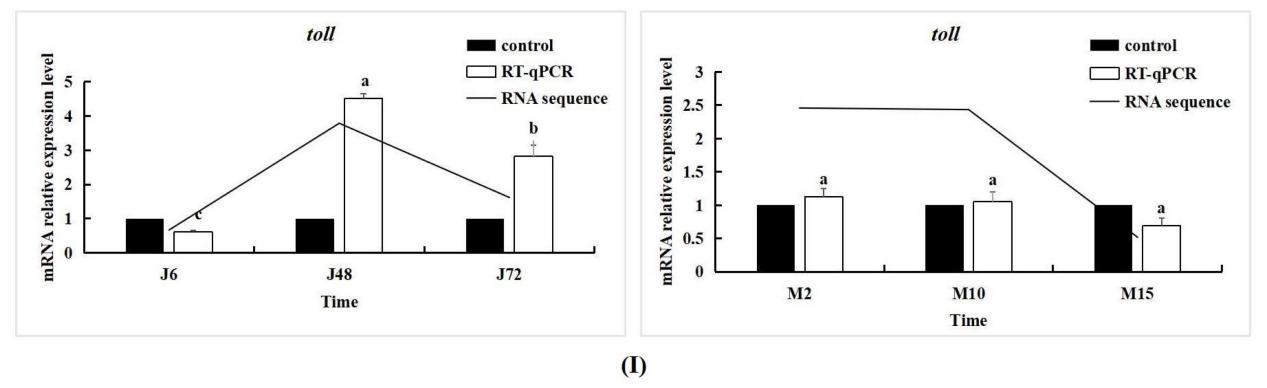


Figure S8. RT-qPCR verification results of DEGs in the hepatopancreas

Note: A: *hsp*70, B: *hsp*90, C: *mincle*, D: *nlrp*12, E: *myd*88, F: *imd*, G: *casp*7, H: *casp*9, I: *toll*."control" indicates the expression of related genes expression under salinity at 20 psu.


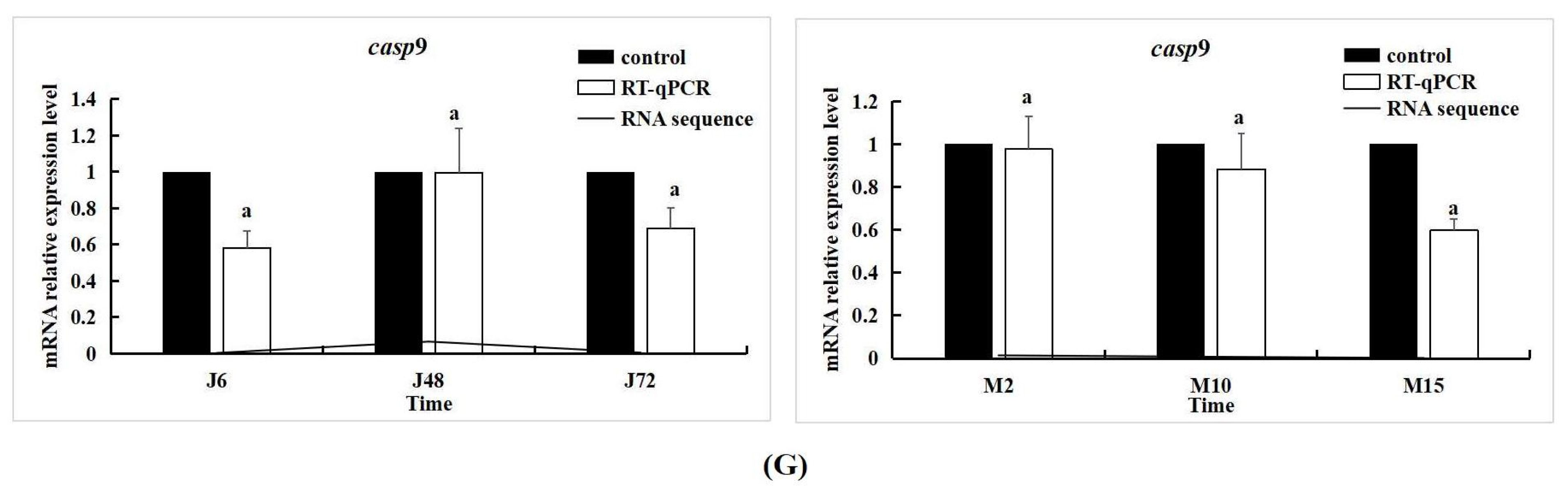

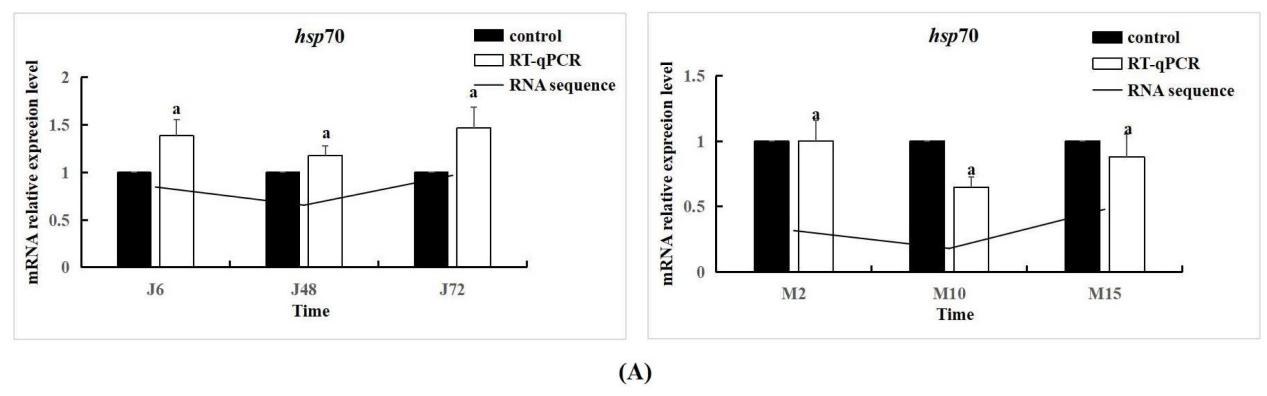

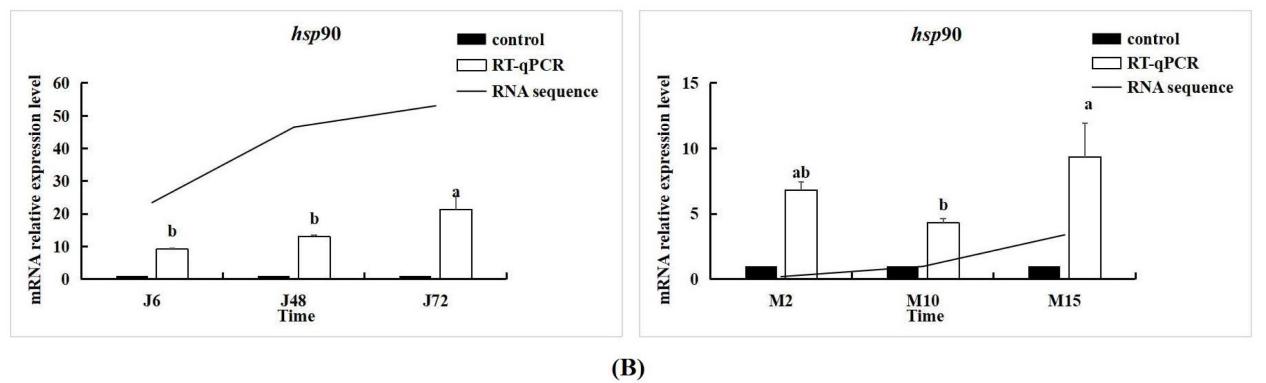

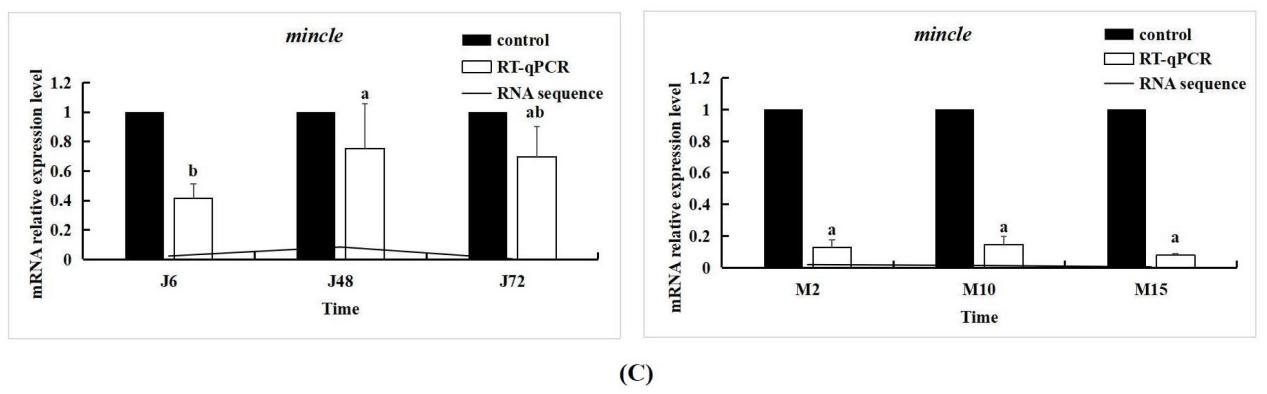

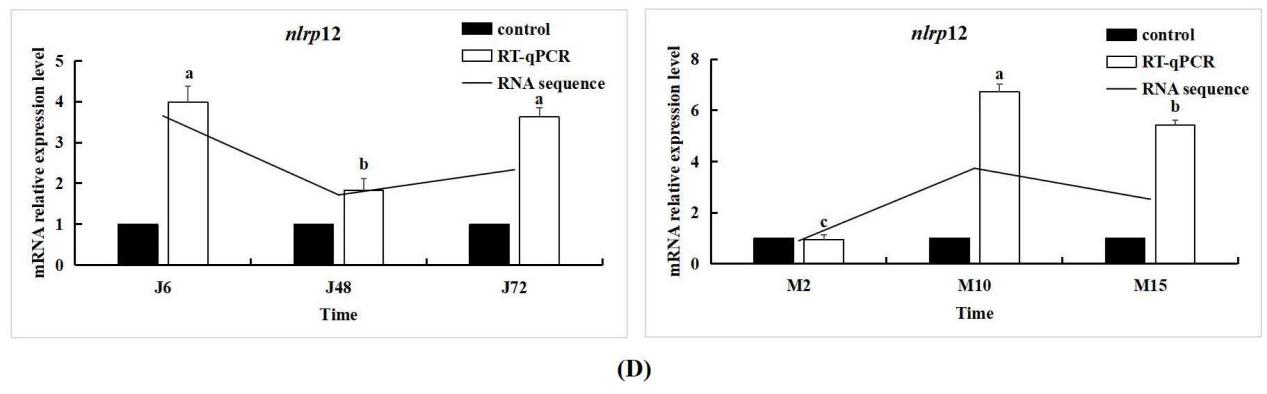

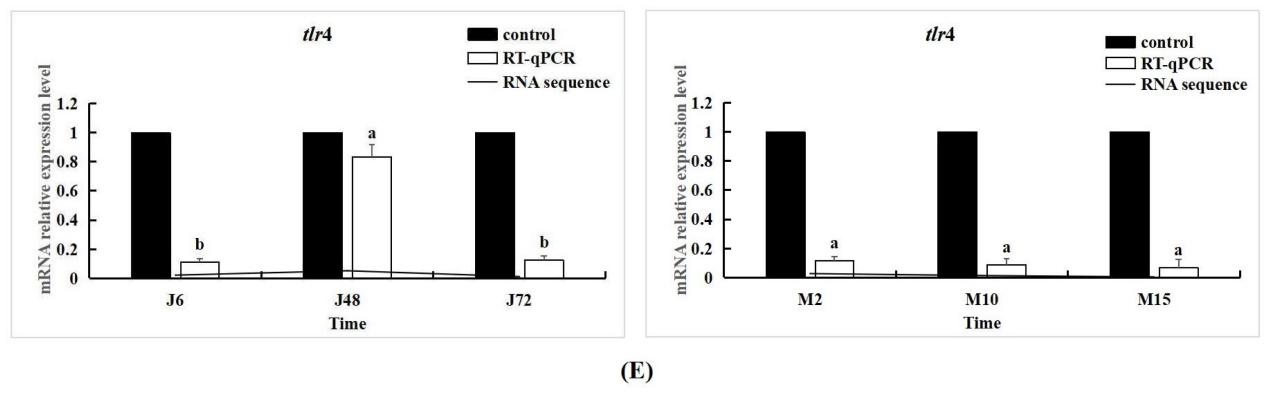

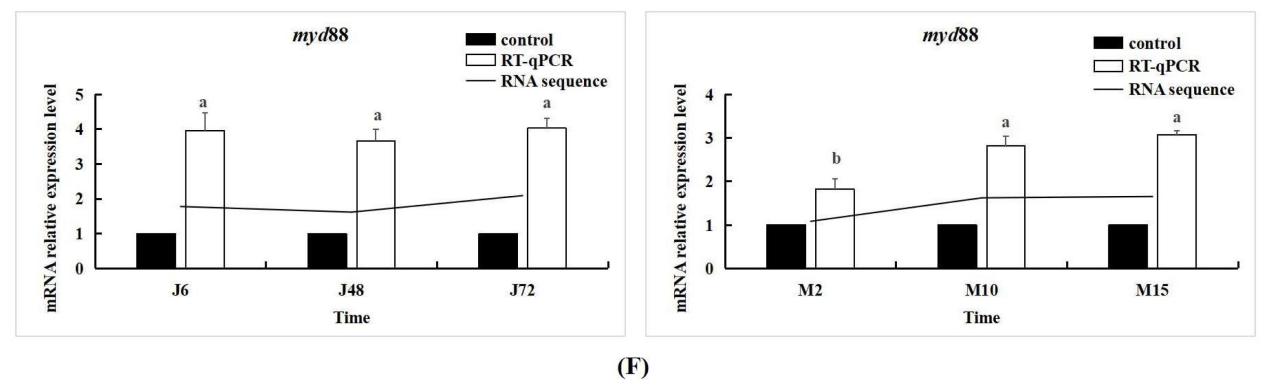


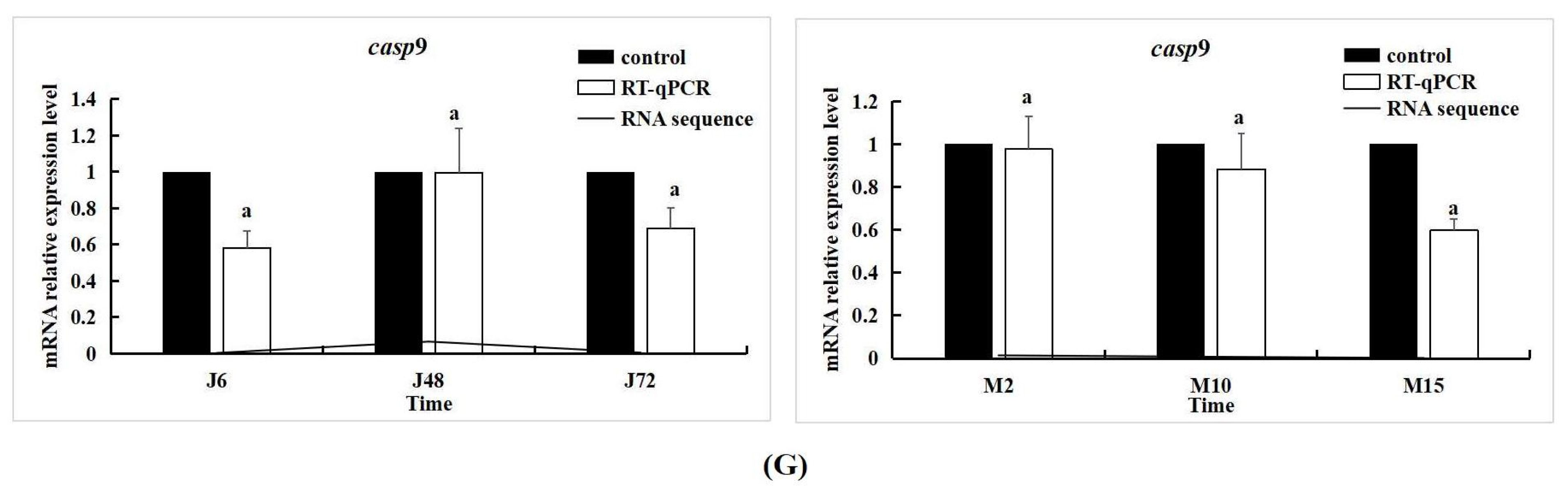


Figure S9. RT-qPCR verification results of DEGs in the hemocytes

Note: A: *hsp*70, B: *hsp*90, C: *mincle*, D: *nlrp*12, E: *trl*4, F: *myd*88, G: *casp*9."control" indicates the expression of related genes expression under salinity at 20 psu.

The data in the figure are the average value and standard error. The ordinate represents the relative RT-qPCR expression of each gene. Different letters on the column signify the significance between the groups (Duncan test, *p < 0.05*). The broken line depicts the trend of change observed in the transcriptome sequencing results of each gene.
